# Supplementary material for: Impact of RBMS 3 Progression on Expression of EMT Markers
Source: Cells. 2024 Sep 14;13(18):1548. doi: 10.3390/cells13181548 (PMC11430492; doi:10.3390/cells13181548)

IHC expression pattern presentation (Magnification x200):

A) RBMS 3

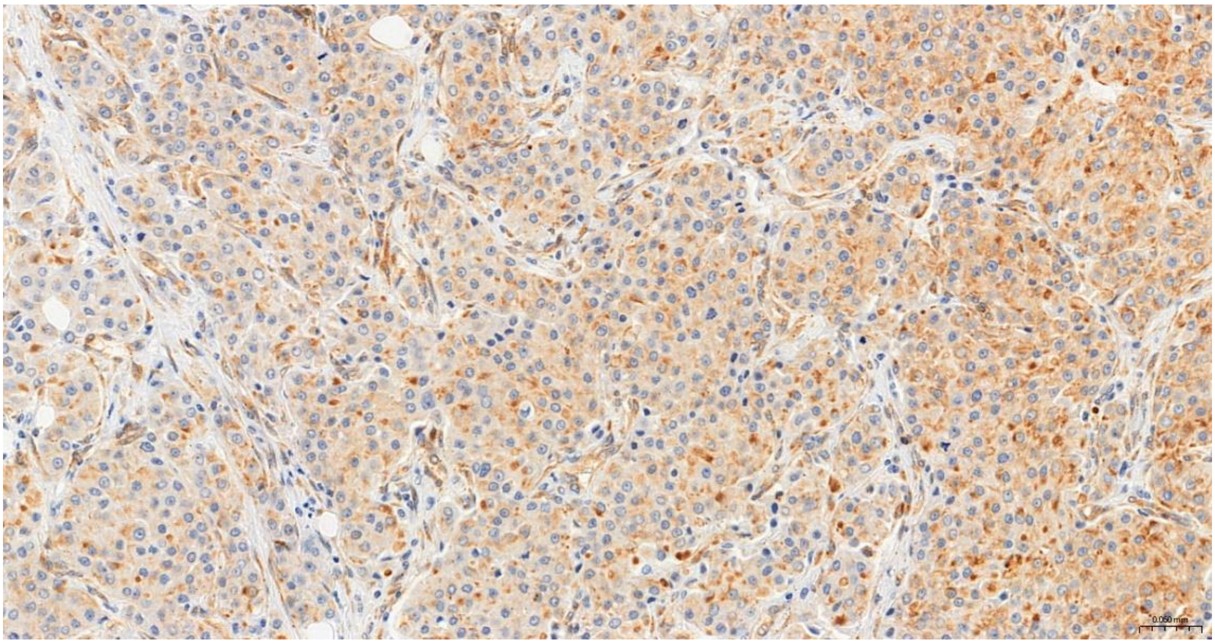

B) TWIST 1

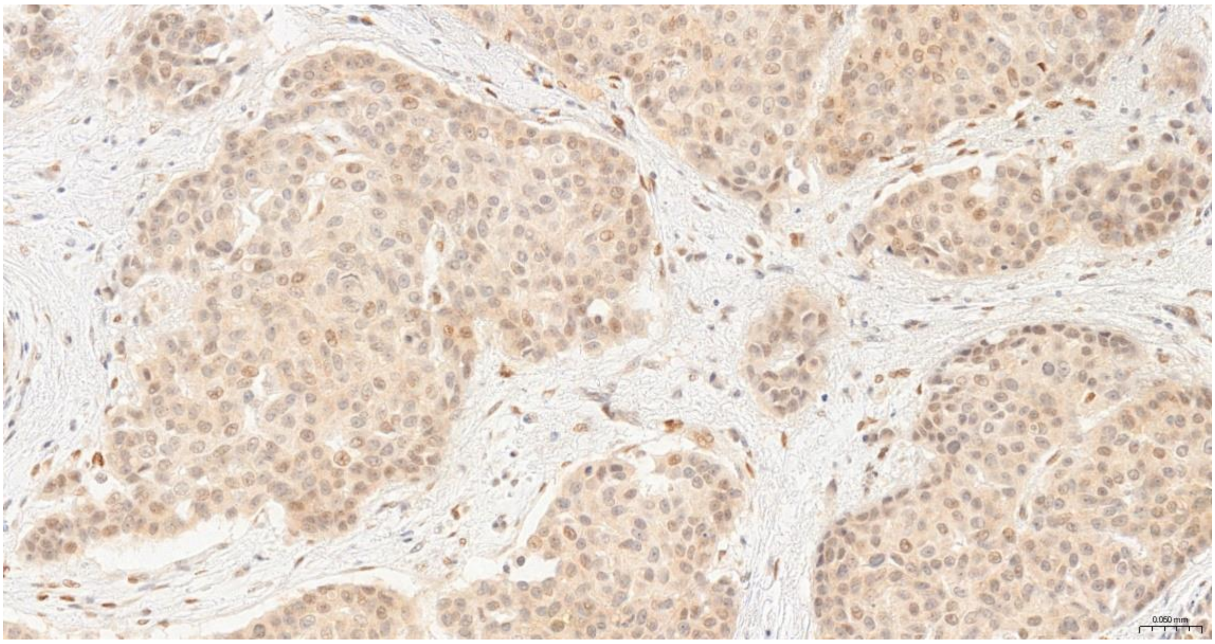

C) SNAIL

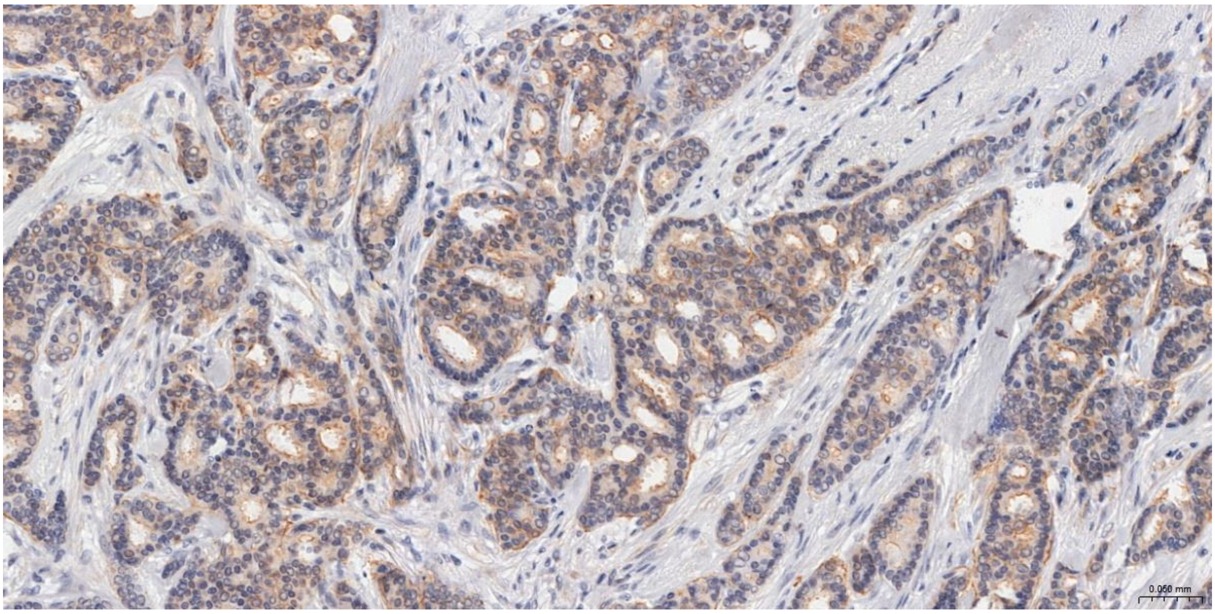

D) SLUG

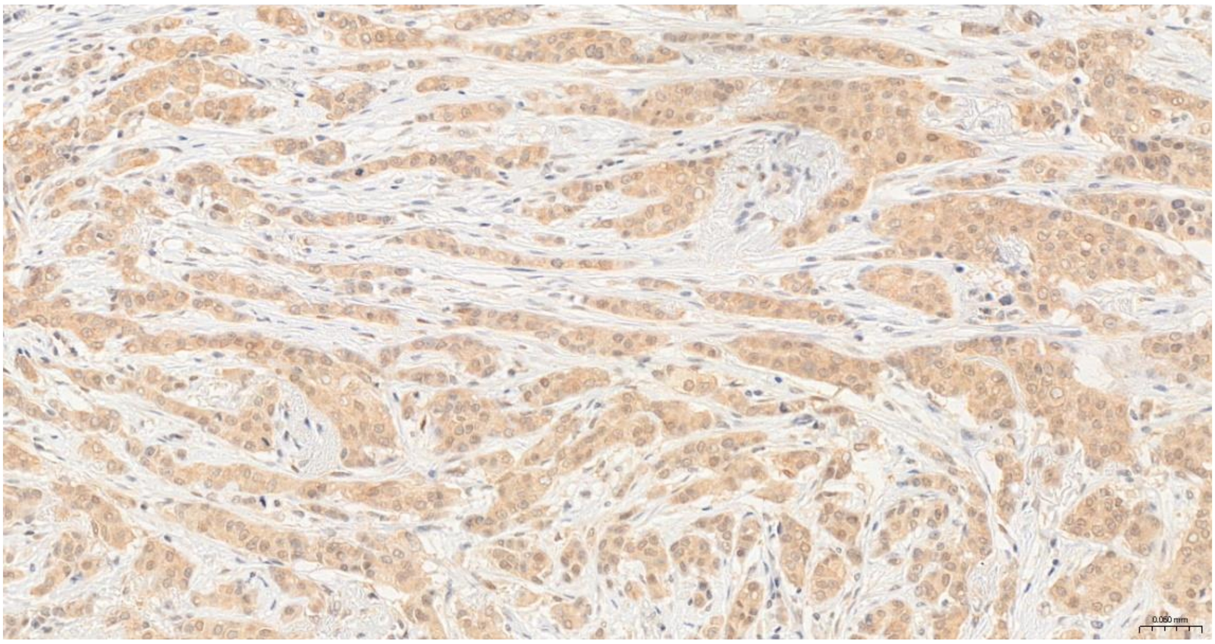

E) E-Cadherin

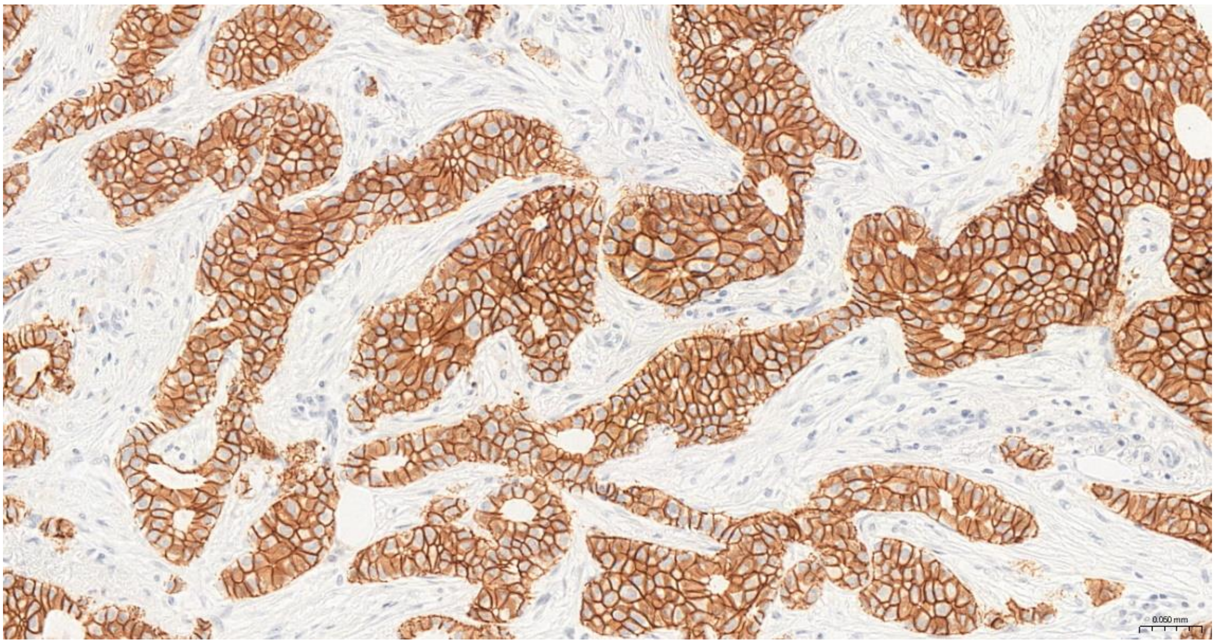

F) N-Cadherin

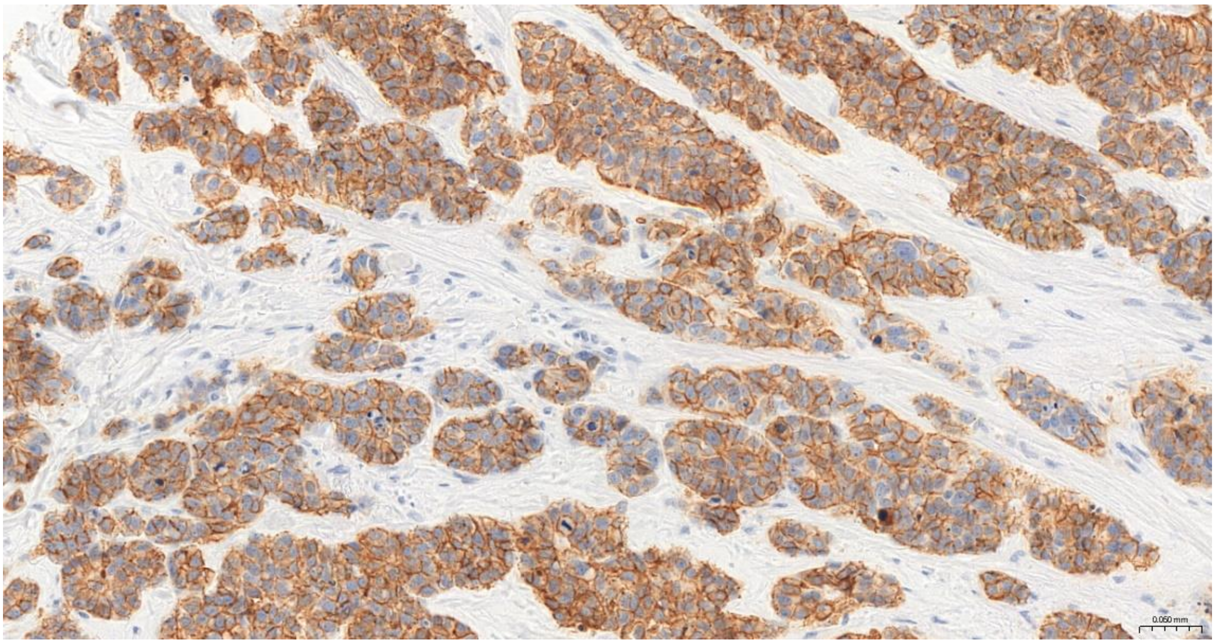

G) ZEB 1

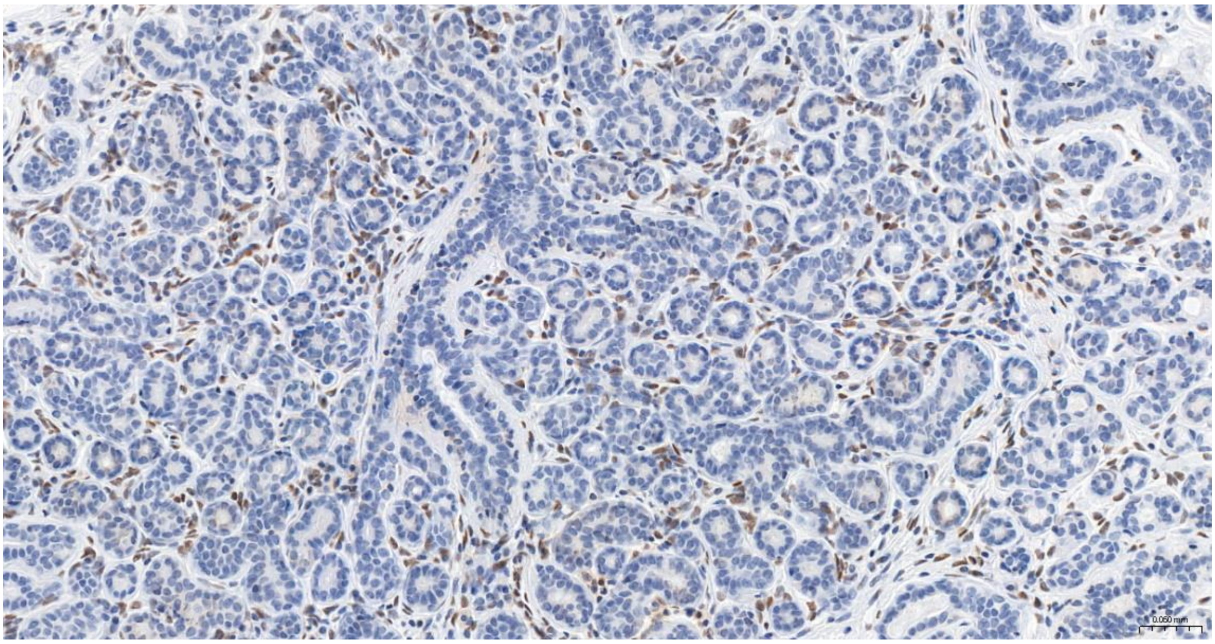

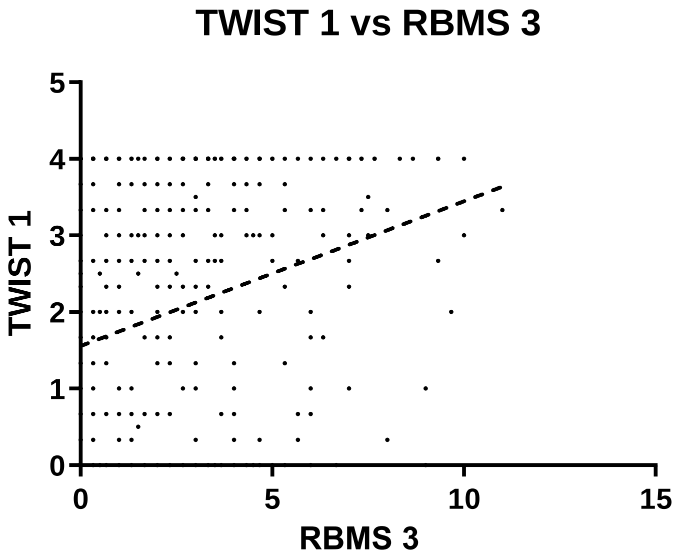

P value	
P (two-tailed)	<0.0001
P value summary	****

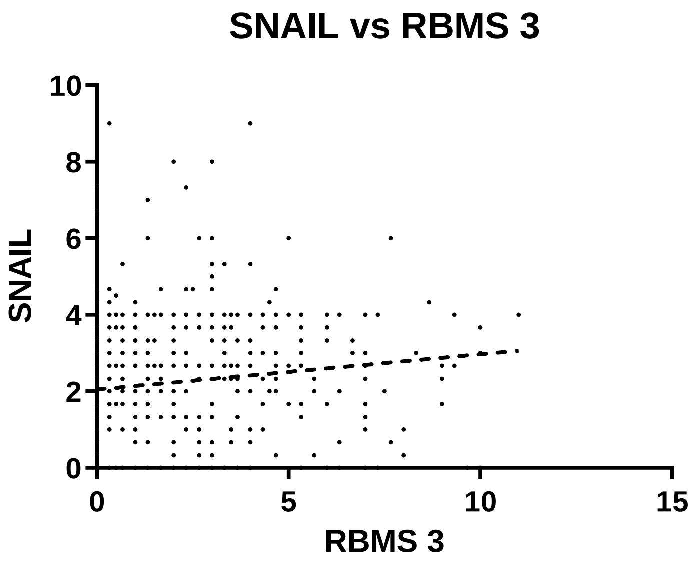

P value	
P (two-tailed)	<0.0001
P value summary	****

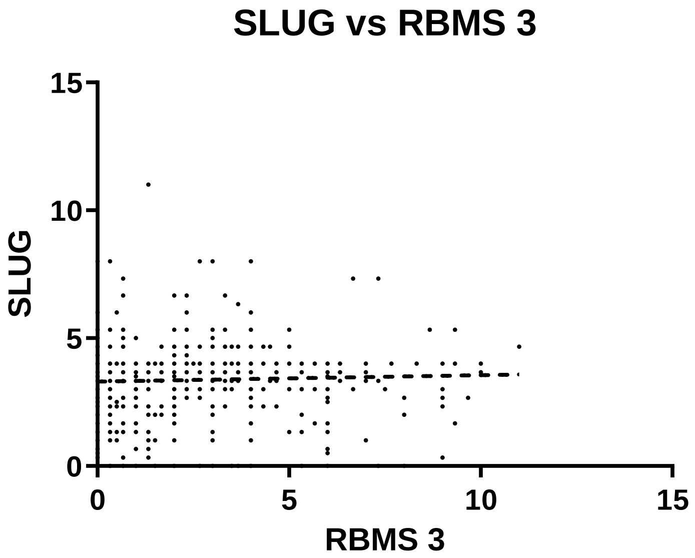

P value	
P (two-tailed)	0.0784
P value summary	ns

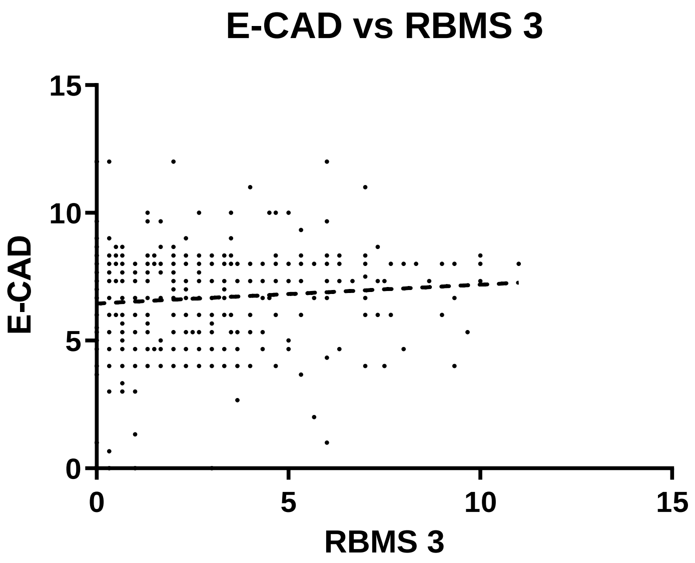

P value	
P (two-tailed)	0.0802
P value summary	ns

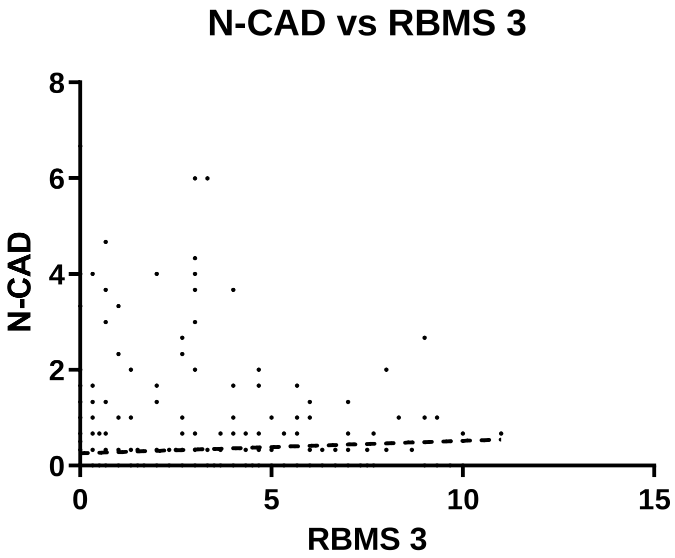

P value	
P (two-tailed)	<0.0001
P value summary	****

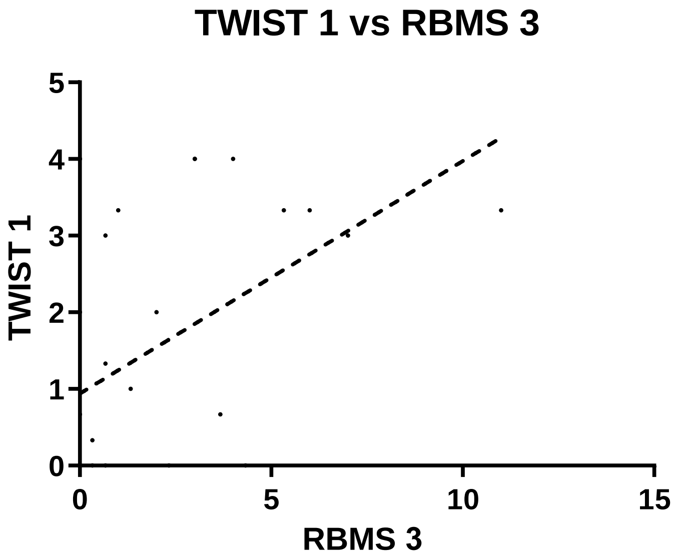

P value	
P (two-tailed)	0.0106
P value summary	*

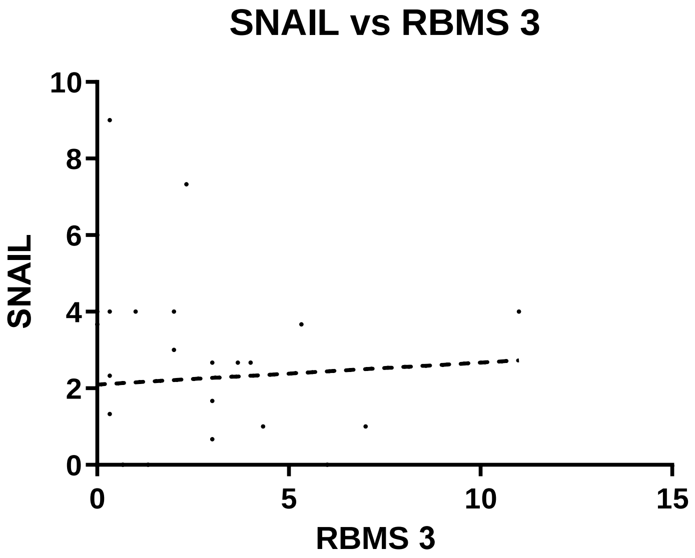

P value	
P (two-tailed)	0.4585
P value summary	ns

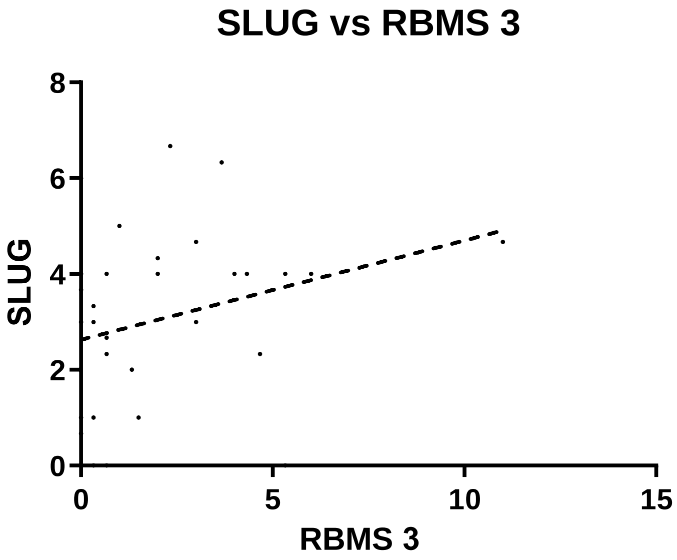

P value	
P (two-tailed)	0.0806
P value summary	ns

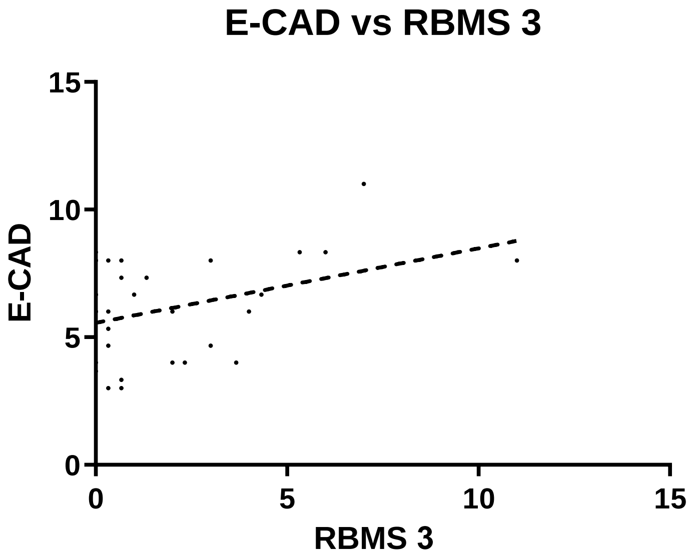

P value	
P (two-tailed)	0.2426
P value summary	ns

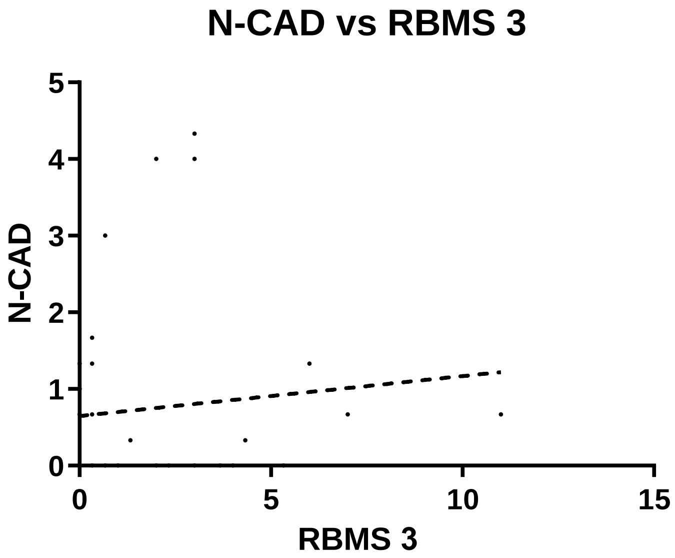

P value	
P (two-tailed)	0.2718
P value summary	ns

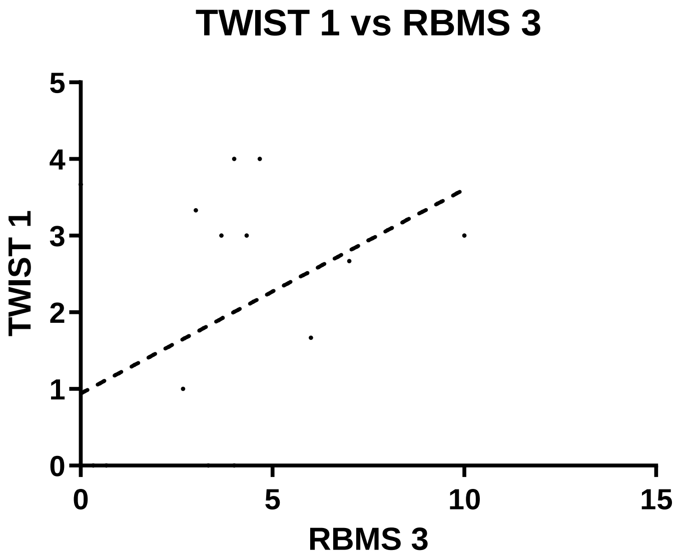

P value	
P (two-tailed)	0.1185
P value summary	ns

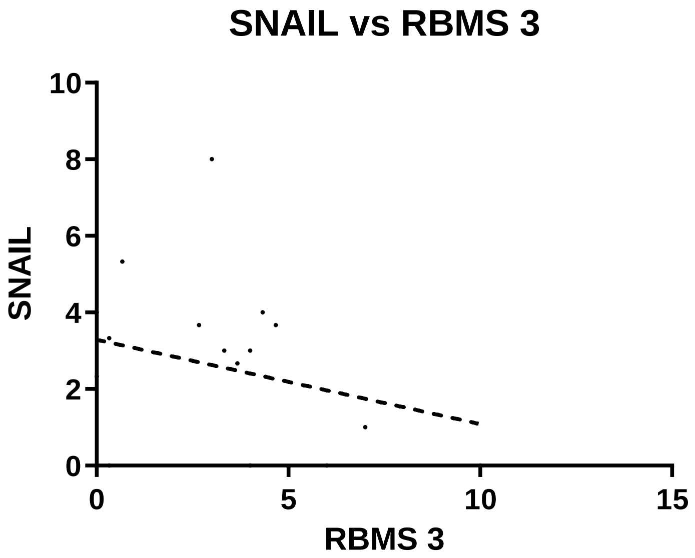

P value	
P (two-tailed)	0.4336
P value summary	ns

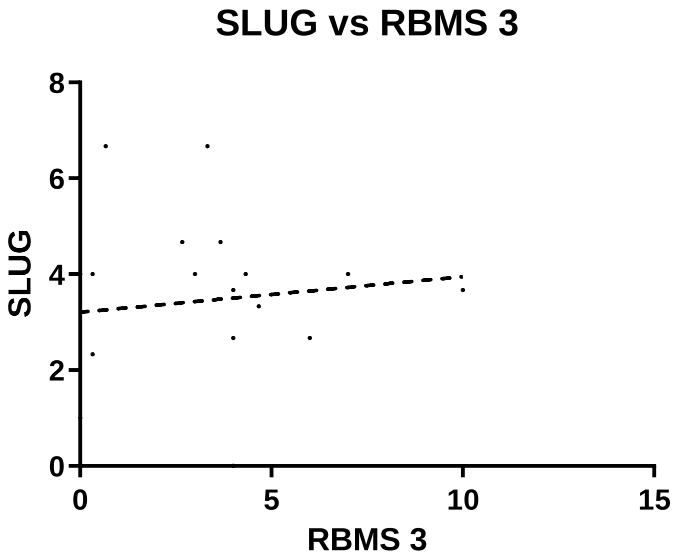

P value	
P (two-tailed)	0.9571
P value summary	ns

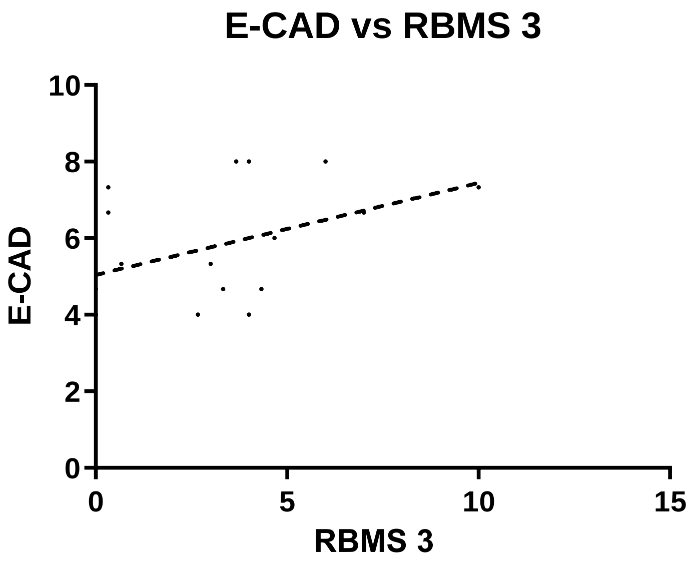

P value	
P (two-tailed)	0.0533
P value summary	ns

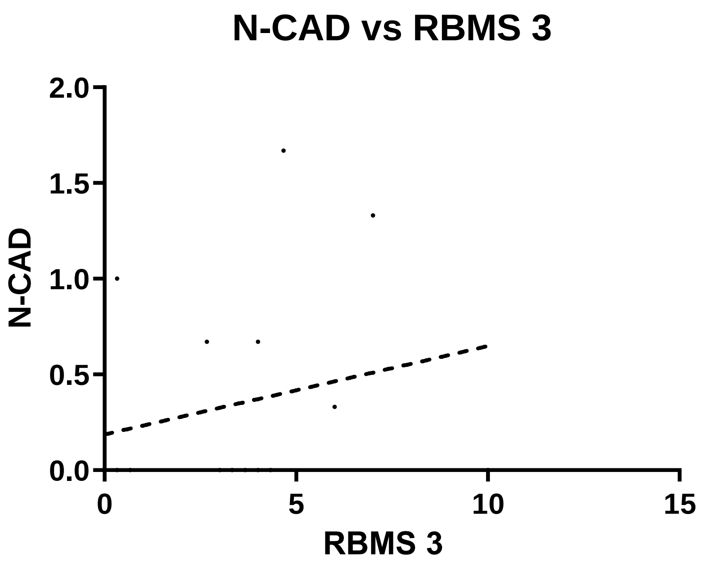

P value	
P (two-tailed)	0.1576
P value summary	ns

G2 TNBC RBMS 3 vs TWIST 1

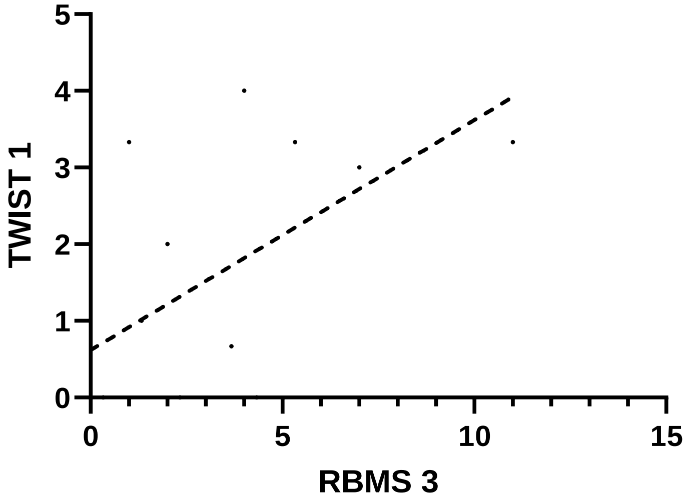

P value	
P (two-tailed)	0.0250
P value summary	*

G2 TNBC RBMS 3 vs SNAIL

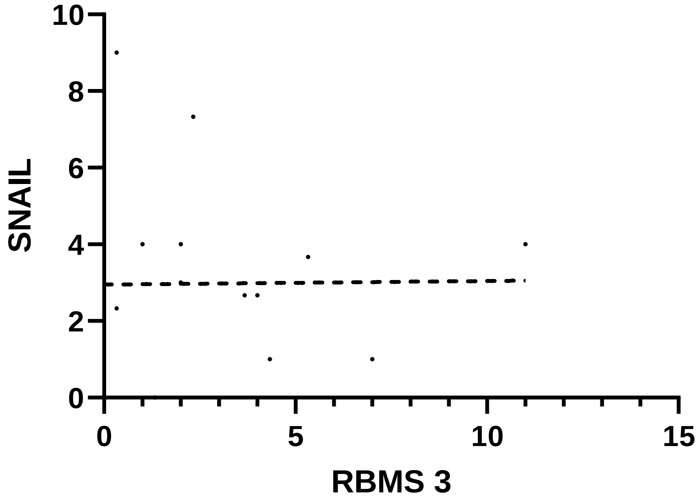

P value	
P (two-tailed)	0.4371
P value summary	ns

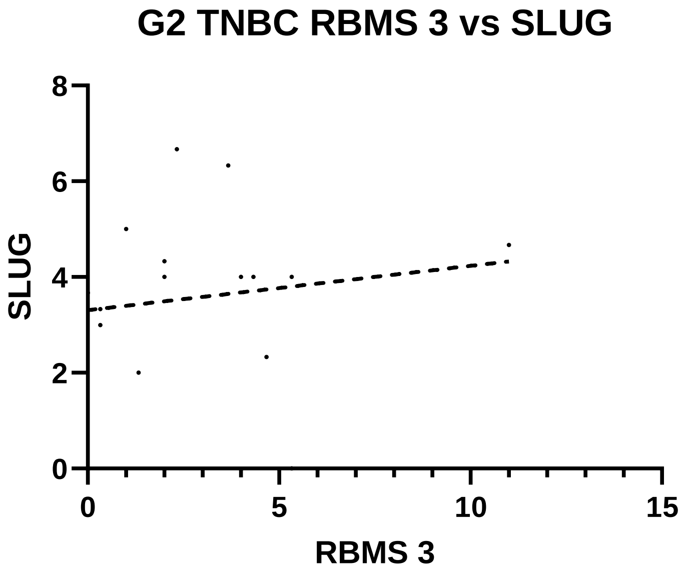

P value	
P (two-tailed)	0.3931
P value summary	ns

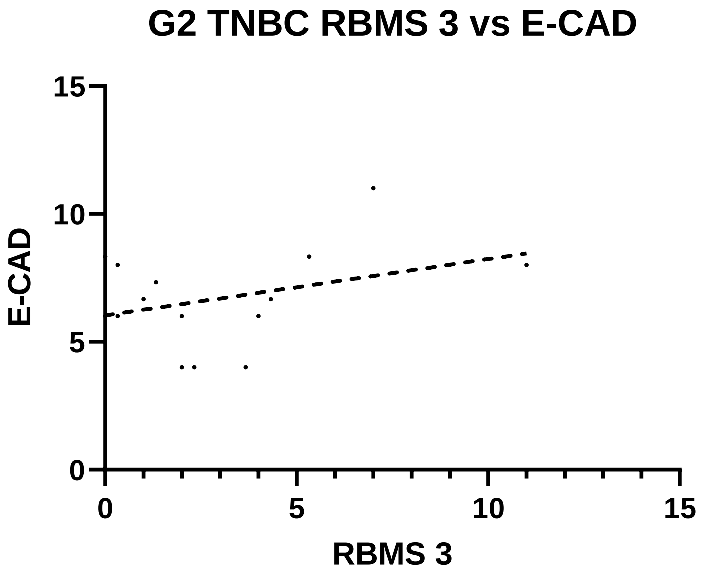

P value	
P (two-tailed)	0.5537
P value summary	ns

G2 TNBC RBMS 3 vs N-CAD

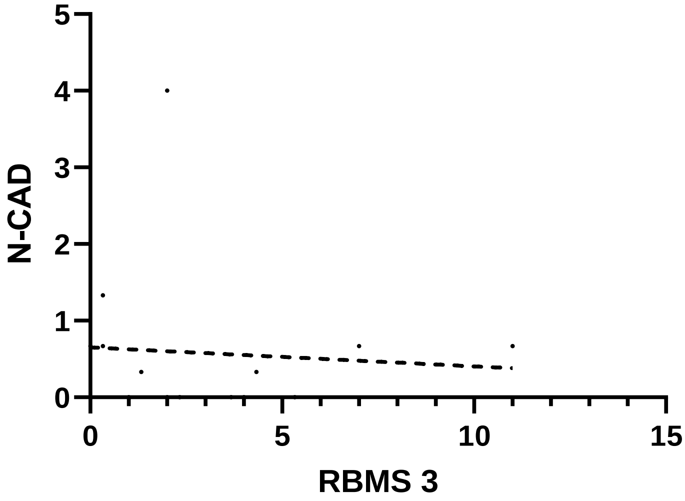

P value	
P (two-tailed)	0.7164
P value summary	ns

G3 TNBC RBMS 3 vs TWIST 1

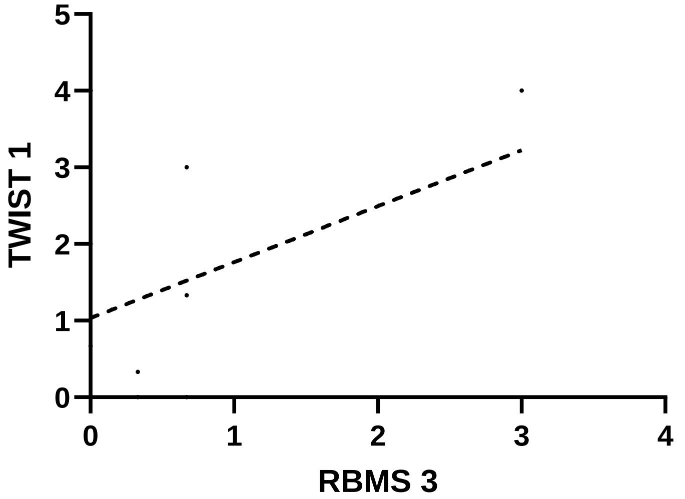

P value	
P (two-tailed)	0.9404
P value summary	ns

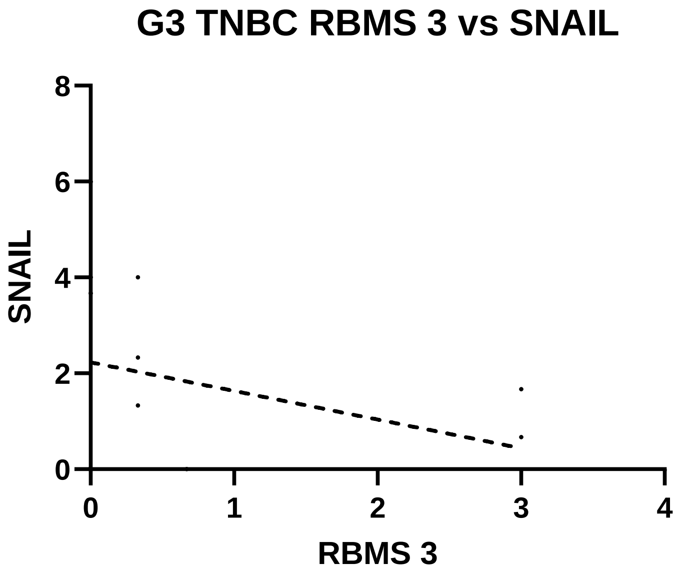

P value	
P (two-tailed)	0.1042
P value summary	ns

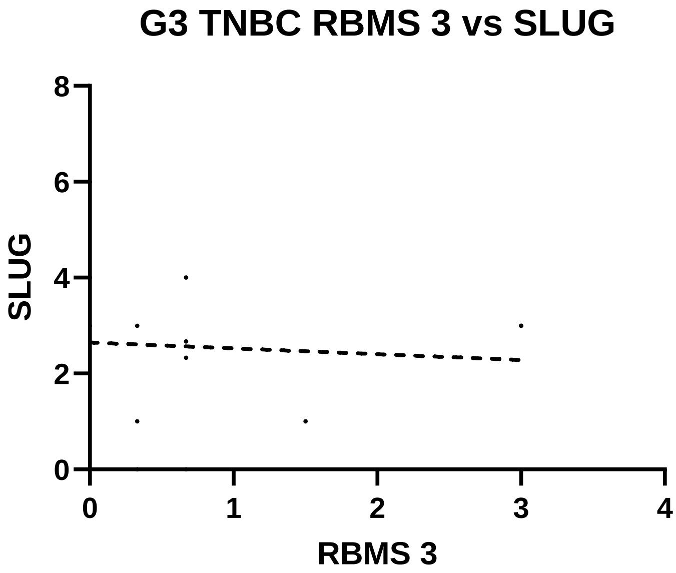

P value	
P (two-tailed)	0.2144
P value summary	ns

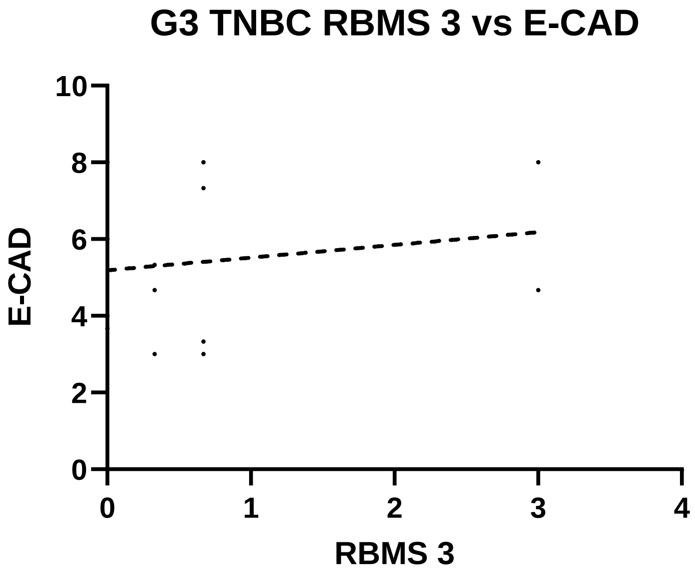

P value	
P (two-tailed)	0.9583
P value summary	ns

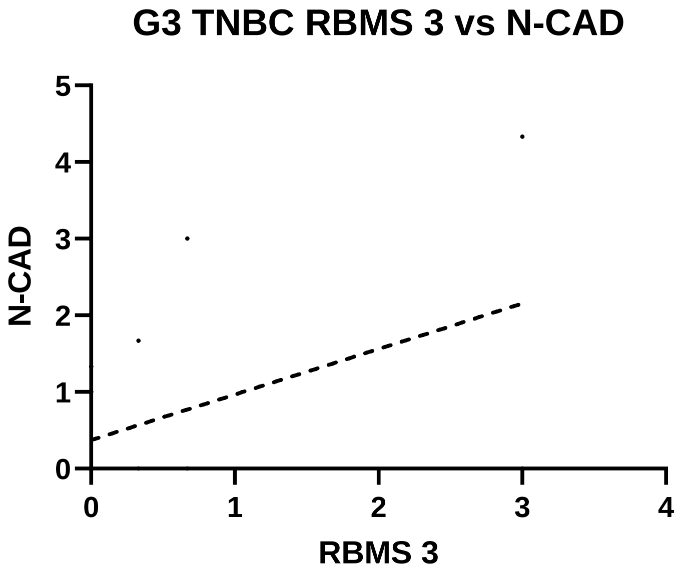

P value	
P (two-tailed)	0.5403
P value summary	ns

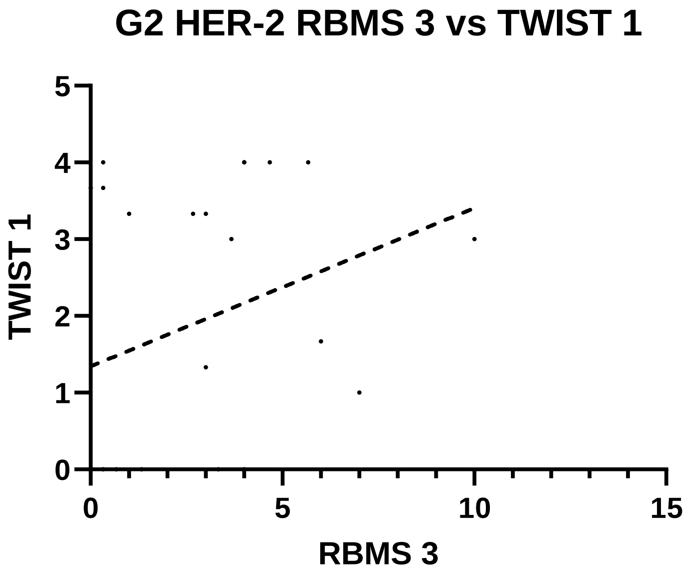

P value	
P (two-tailed)	0.0927
P value summary	ns

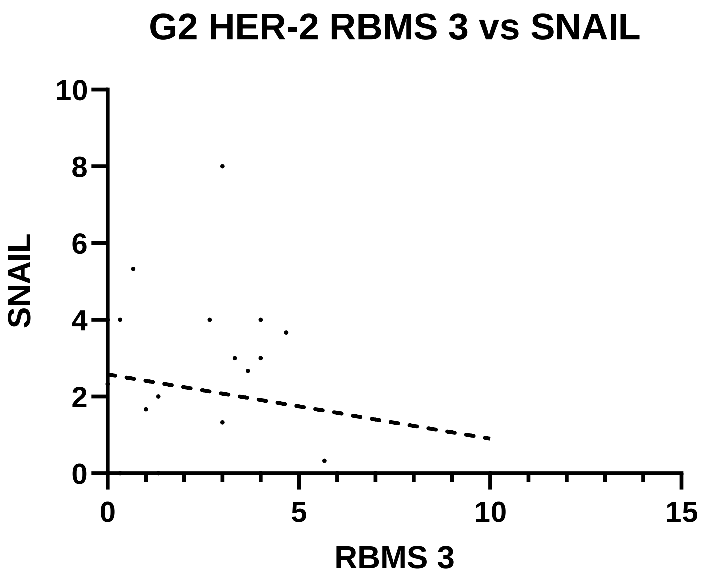

P value	
P (two-tailed)	0.4118
P value summary	ns

G2 HER-2 RBMS 3 vs SLUG

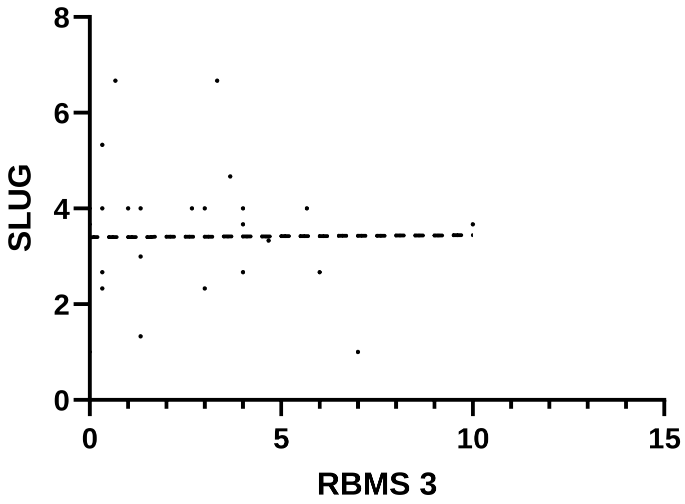

P value	
P (two-tailed)	0.8762
P value summary	ns

G2 HER-2 RBMS 3 vs E-CAD

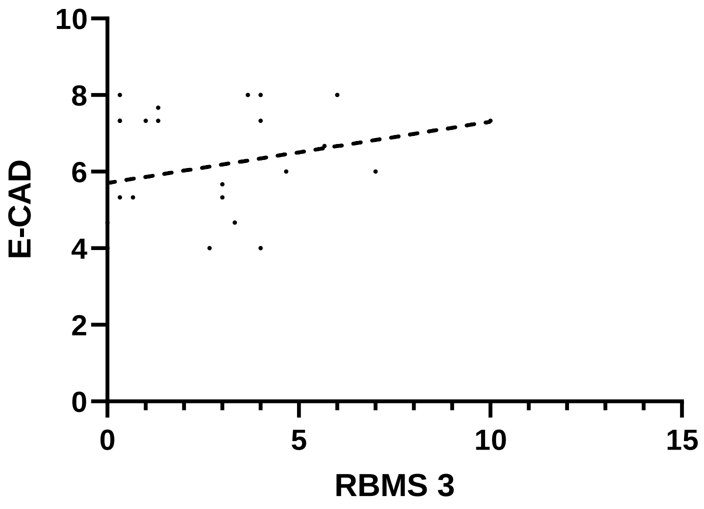

P value	
P (two-tailed)	0.0711
P value summary	ns

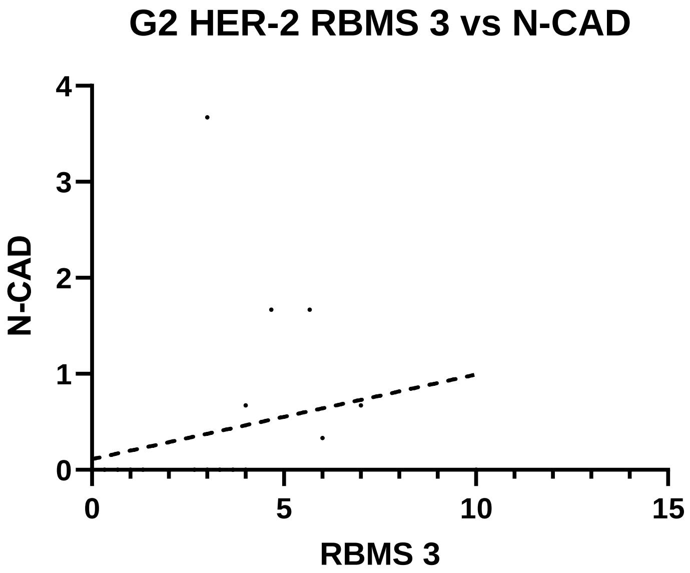

P value	
P (two-tailed)	0.0032
P value summary	**

G3 HER-2 RBMS 3 vs TWIST 1

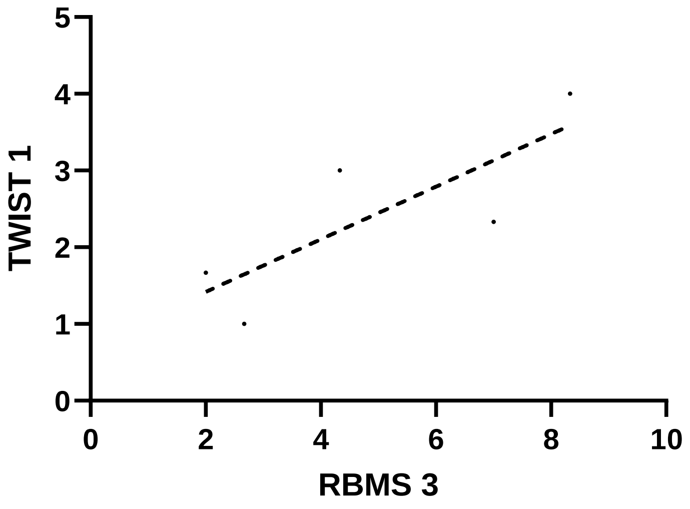

P value	
P (two-tailed)	0.1333
P value summary	ns

G3 HER-2 RBMS 3 vs SNAIL

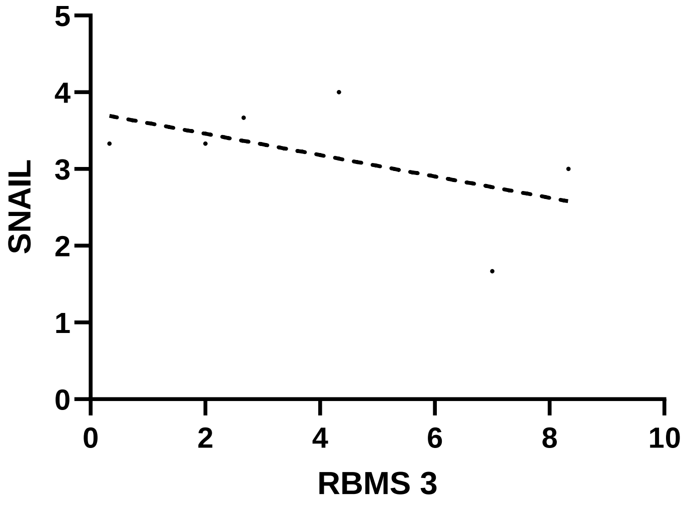

P value	
P (two-tailed)	0.4444
P value summary	ns

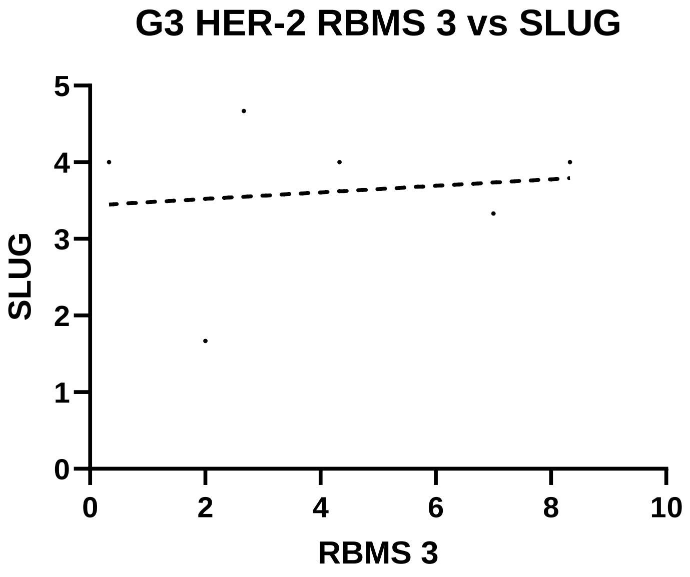

P value	
P (two-tailed)	>0.9999
P value summary	ns

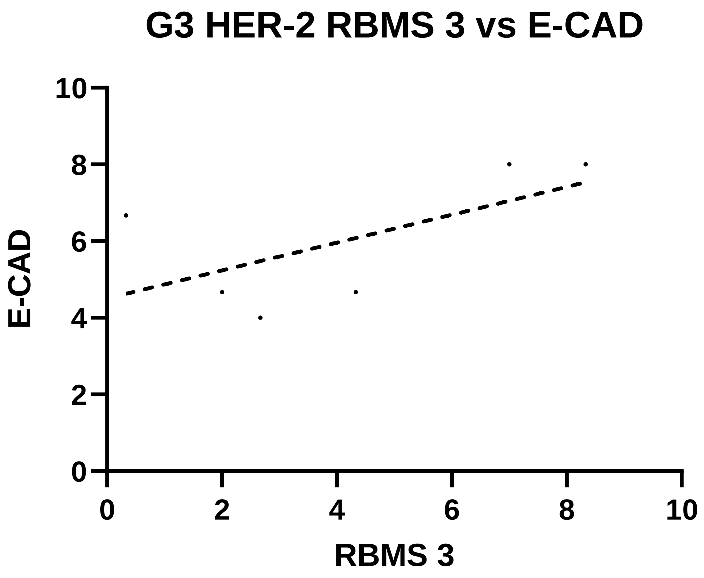

P value	
P (two-tailed)	0.3111
P value summary	ns

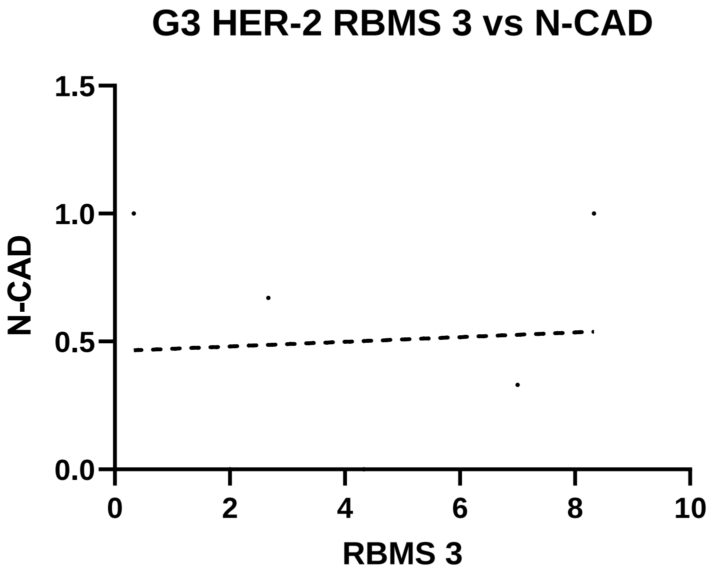

P value	
P (two-tailed)	0.9111
P value summary	ns

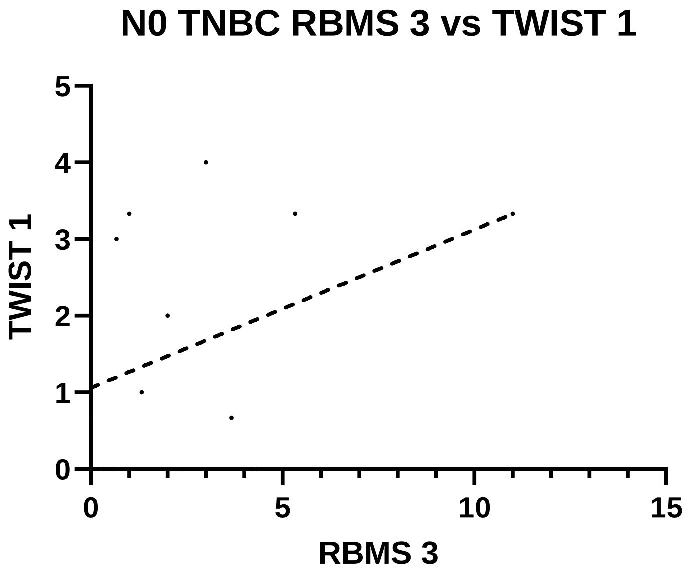

P value	
P (two-tailed)	0.2006
P value summary	ns

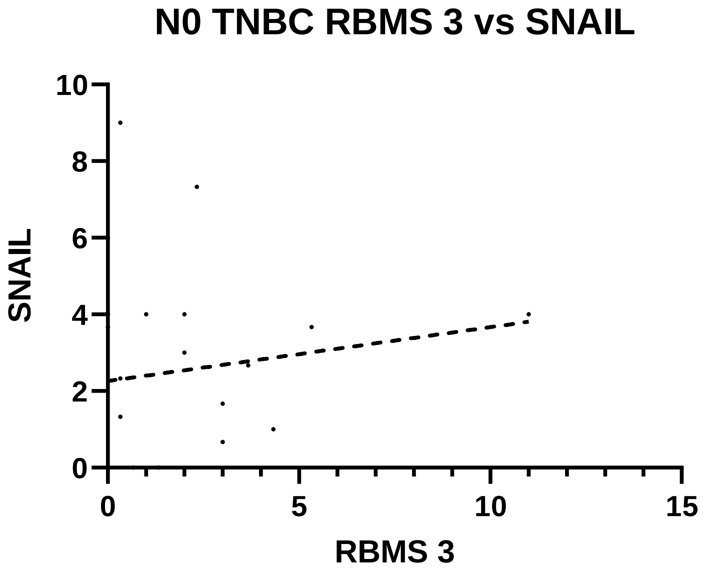

P value	
P (two-tailed)	0.3336

N0 TNBC RBMS 3 vs SLUG

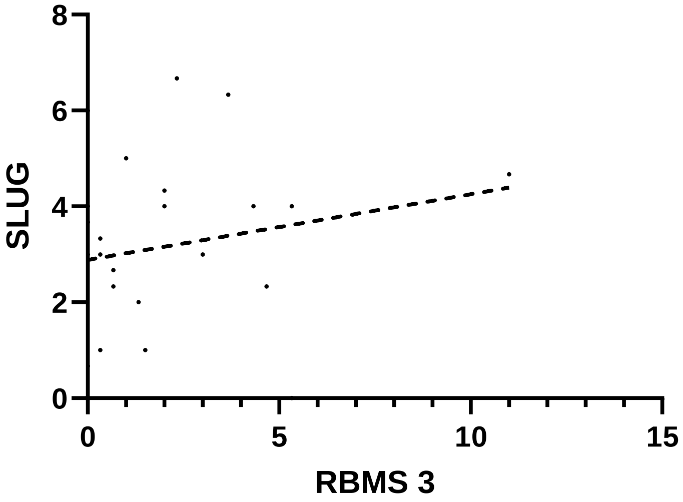

P value	
P (two-tailed)	0.3334
P value summary	ns

N0 TNBC RBMS 3 vs E-CAD

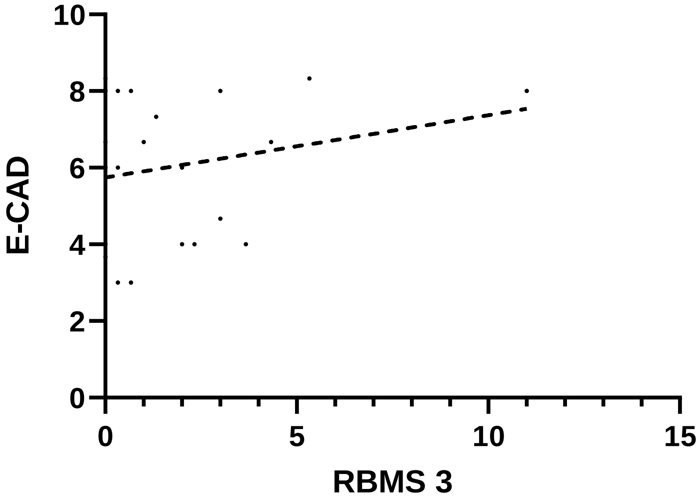

P value	
P (two-tailed)	0.6142
P value summary	ns

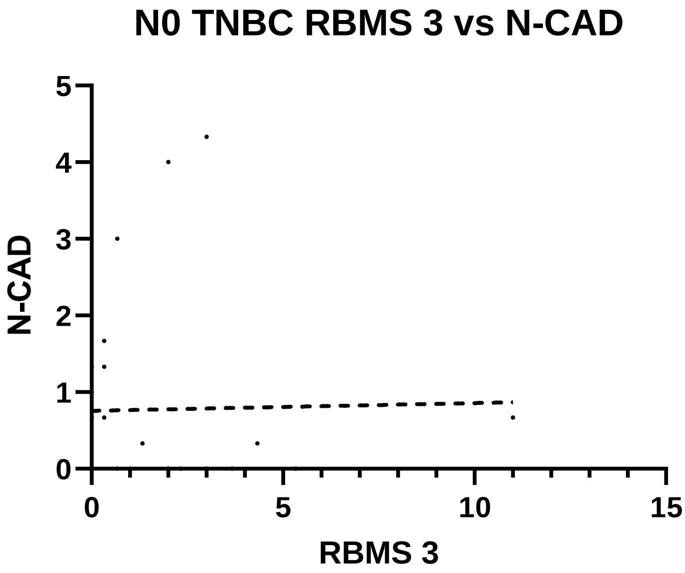

P value	
P (two-tailed)	0.7586
P value summary	ns

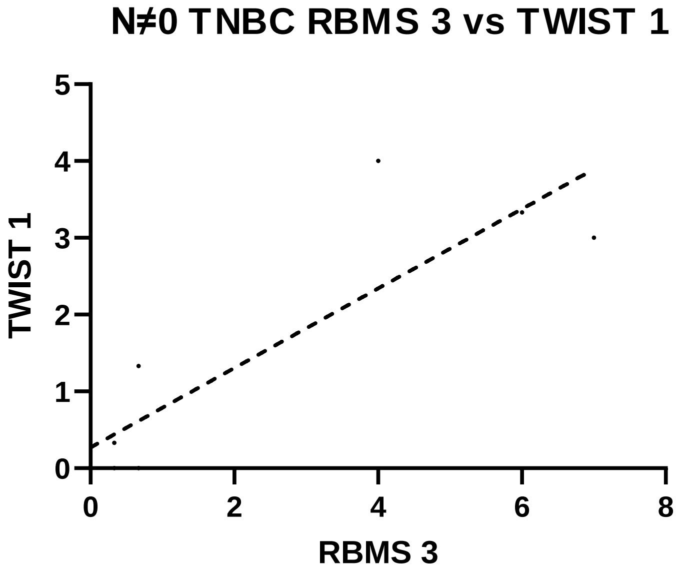

P value	
P (two-tailed)	0.0315
P value summary	*

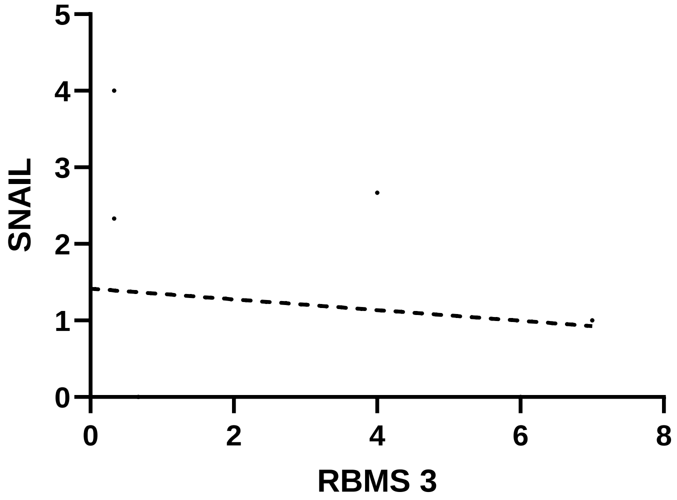

P value	
P (two-tailed)	0.8774
P value summary	ns

N≠0 TNBC RBMS 3 vs SLUG

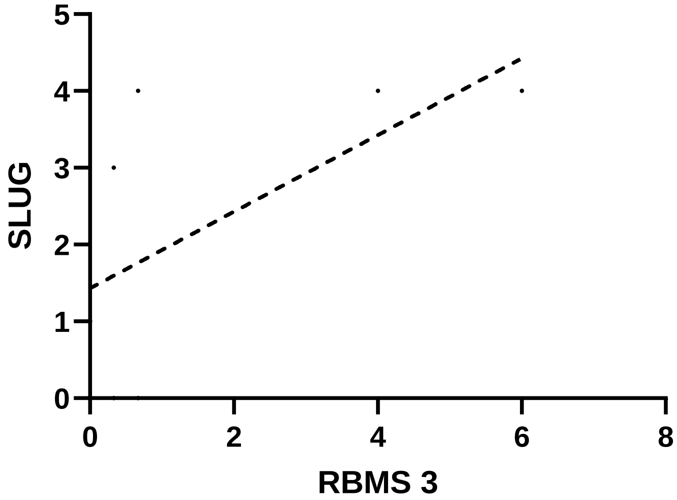

P value	
P (two-tailed)	0.1476
P value summary	ns

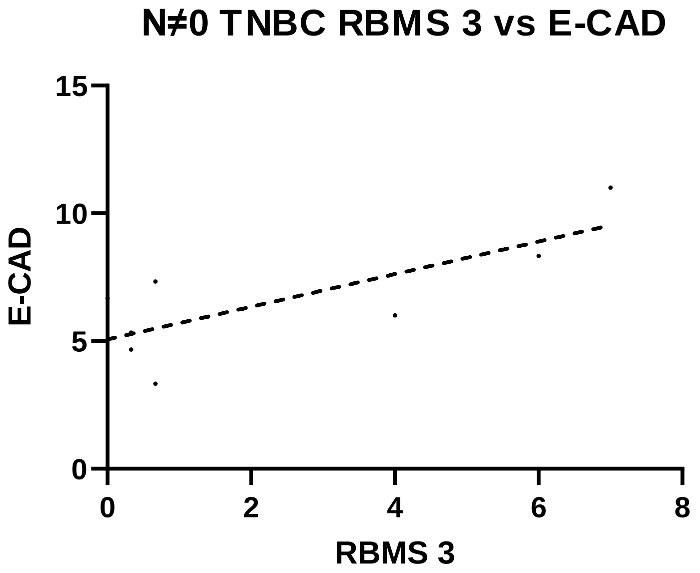

P value	
P (two-tailed)	0.1419
P value summary	ns

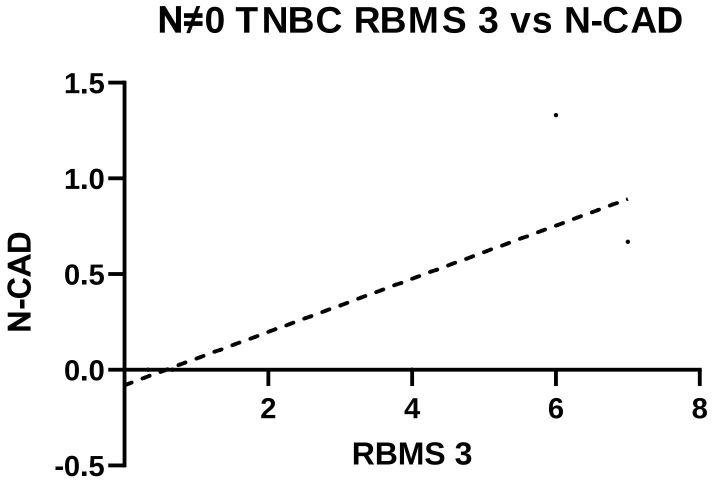

P value	
P (two-tailed)	0.0357
P value summary	*

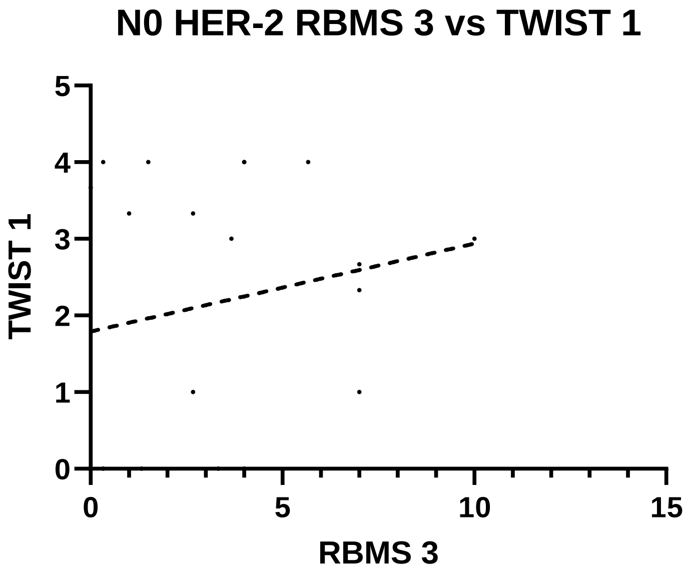

P value	
P (two-tailed)	0.4766
P value summary	ns

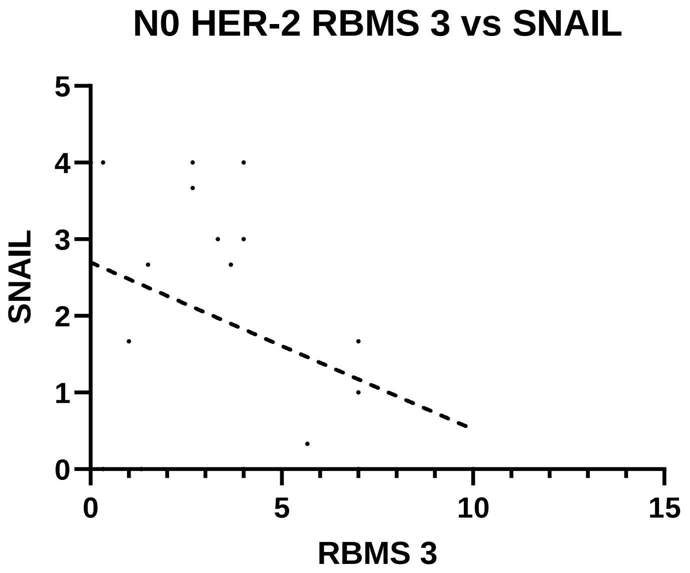

P value	
P (two-tailed)	0.1718
P value summary	ns

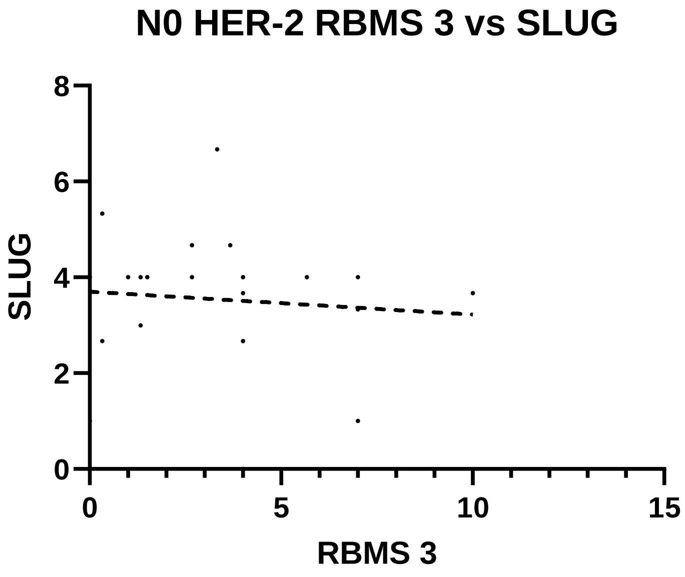

P value	
P (two-tailed)	0.5746
P value summary	ns

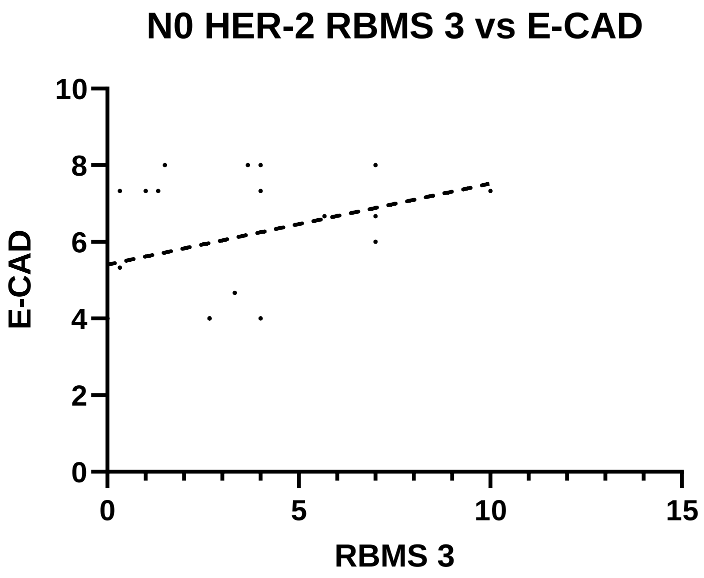

P value	
P (two-tailed)	0.0963
P value summary	ns

N0 HER-2 RBMS 3 vs N-CAD

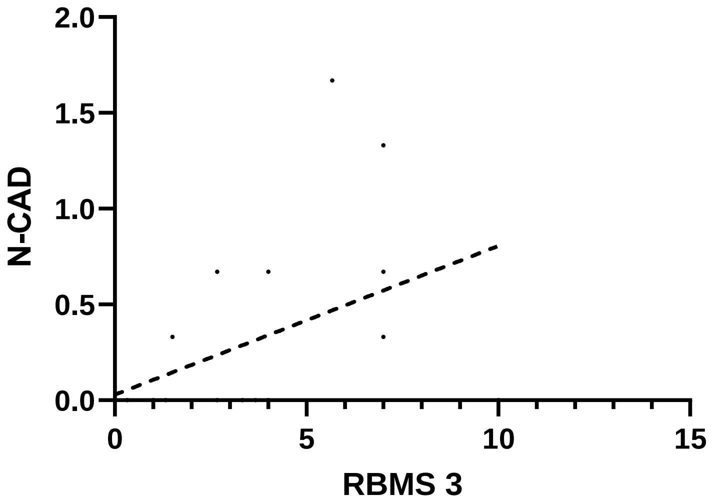

P value	
P (two-tailed)	0.0158
P value summary	*

N≠0 HER-2 RBMS 3 vs TWIST 1

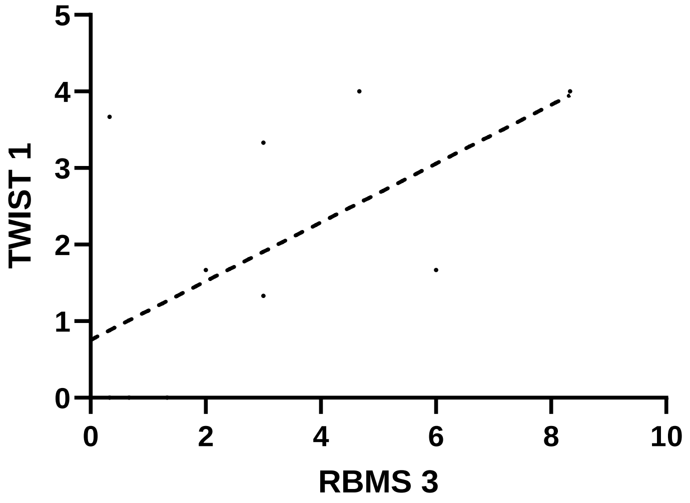

P value	
P (two-tailed)	0.0351
P value summary	*

N≠0 HER-2 RBMS 3 vs SNAIL

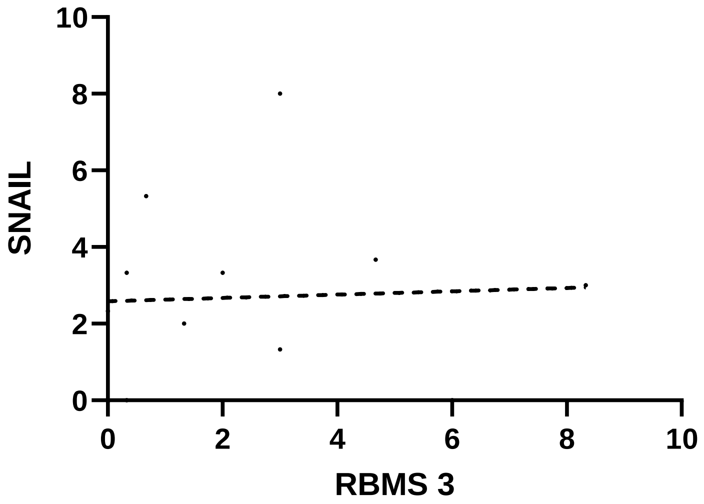

P value	
P (two-tailed)	0.6072
P value summary	ns

N≠0 HER-2 RBMS 3 vs SLUG

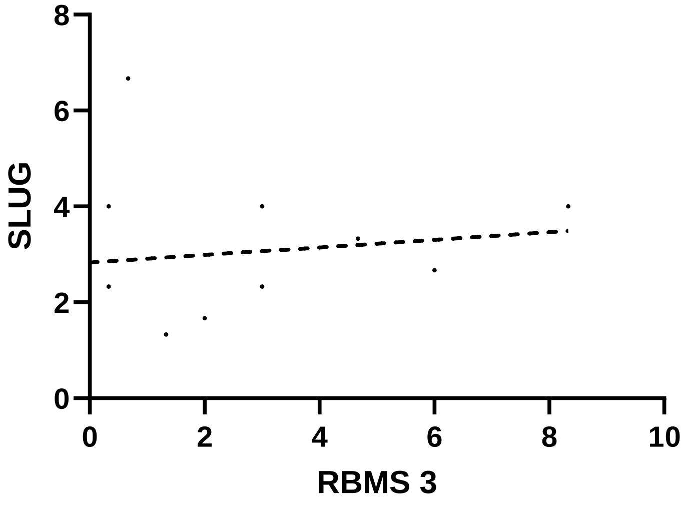

P value	
P (two-tailed)	0.5478
P value summary	ns

N≠0 HER-2 RBMS 3 vs E-CAD

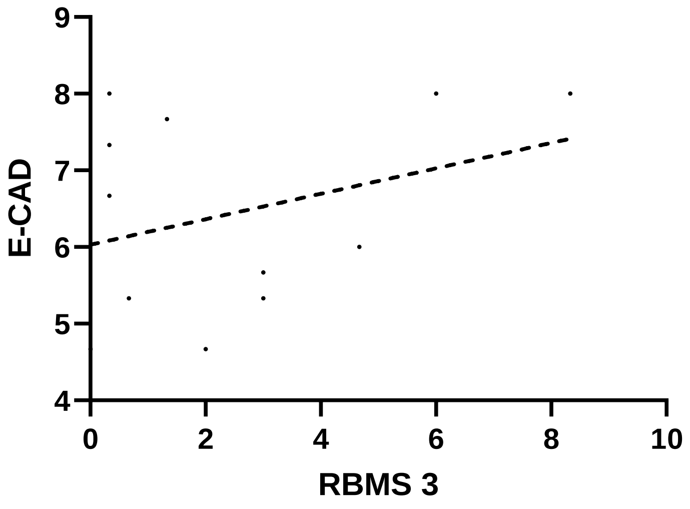

P value	
P (two-tailed)	0.3667
P value summary	ns

N≠0 HER-2 RBMS 3 vs N-CAD

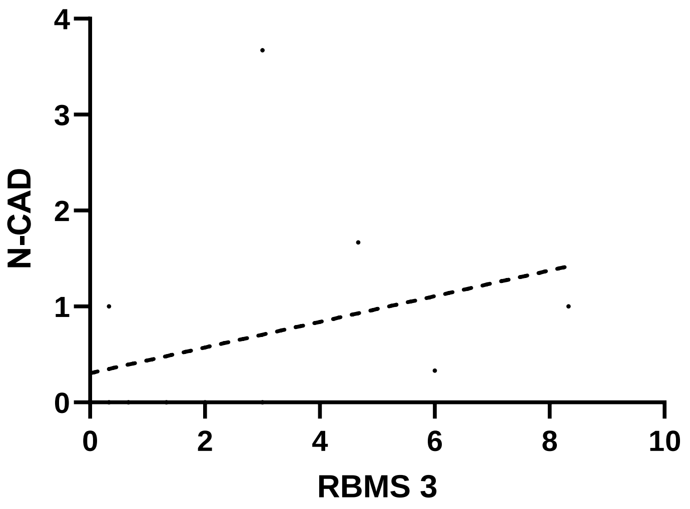

P value	
P (two-tailed)	0.0687
P value summary	ns

1. **RT-qPCR results for RBMS 3 Overexpression**

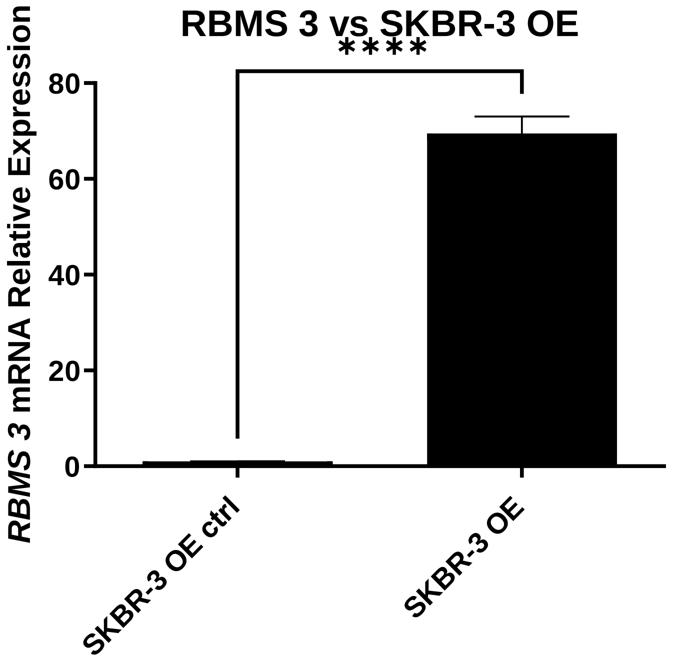

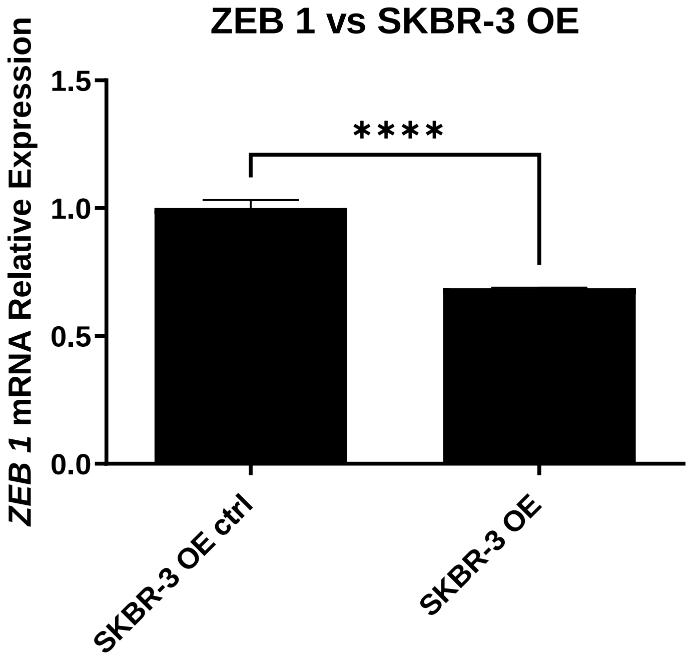

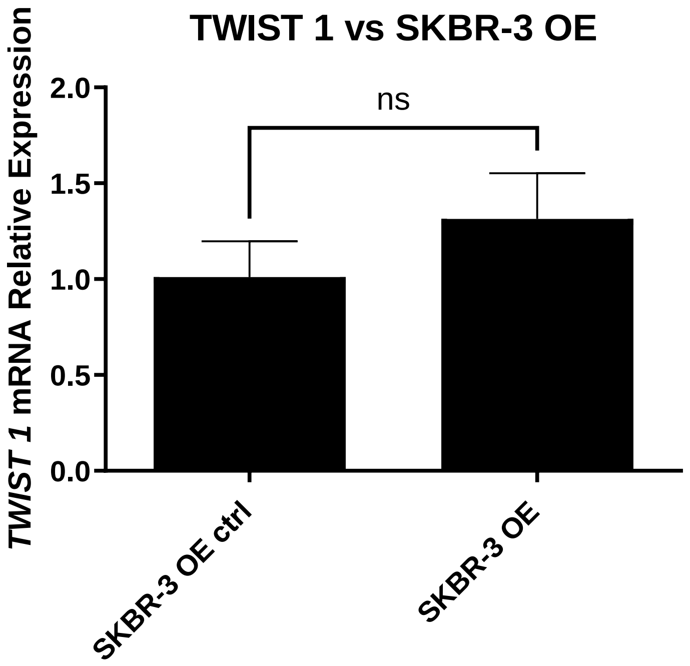
TWIST 1 vs SKBR-3 OE
TWIST 1 mRNA Relative Expression
2.0
1.5
1.0
0.5
0.0
ns
SKBR-3 OE ctrl
SKBR-3 OE

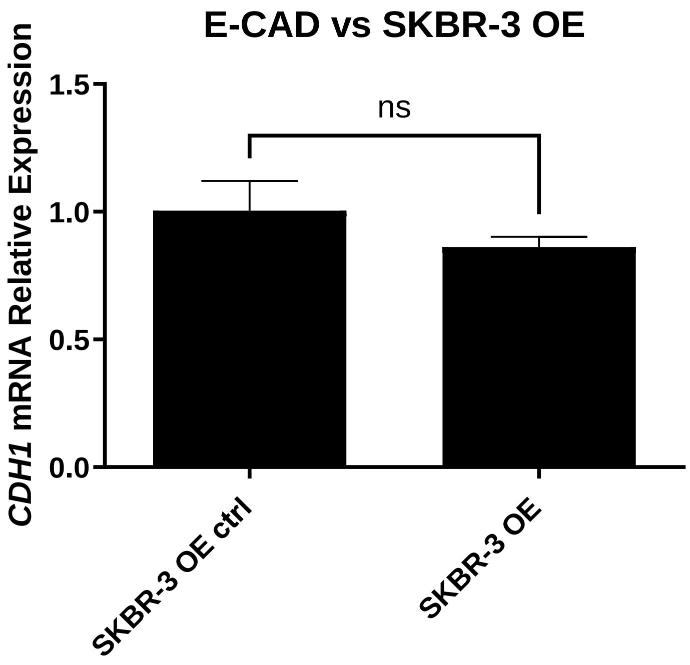
E-CAD vs SKBR-3 OE
CDH1 mRNA Relative Expression
1.5
1.0
0.5
0.0
ns
SKBR-3 OE ctrl
SKBR-3 OE

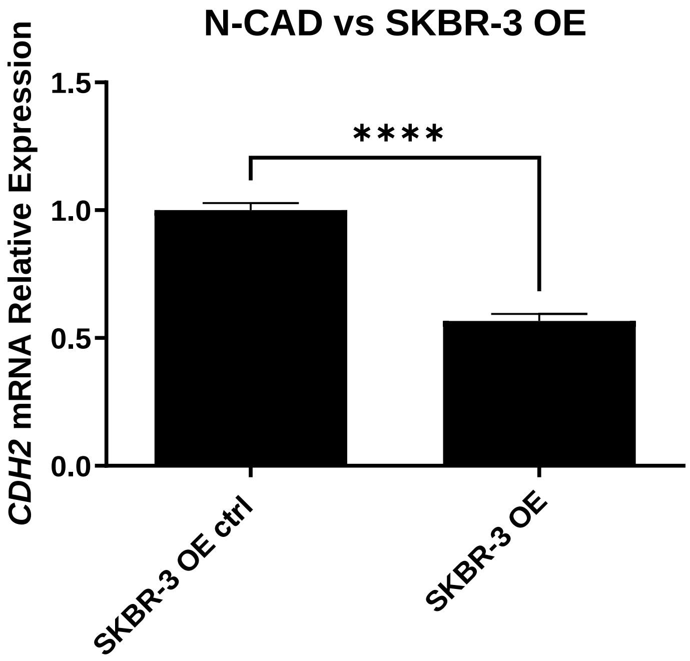

N-CAD vs SKBR-3 OE
CDH2 mRNA Relative Expression
1.5
1.0
0.5
0.0
****
SKBR-3 OE ctrl
SKBR-3 OE

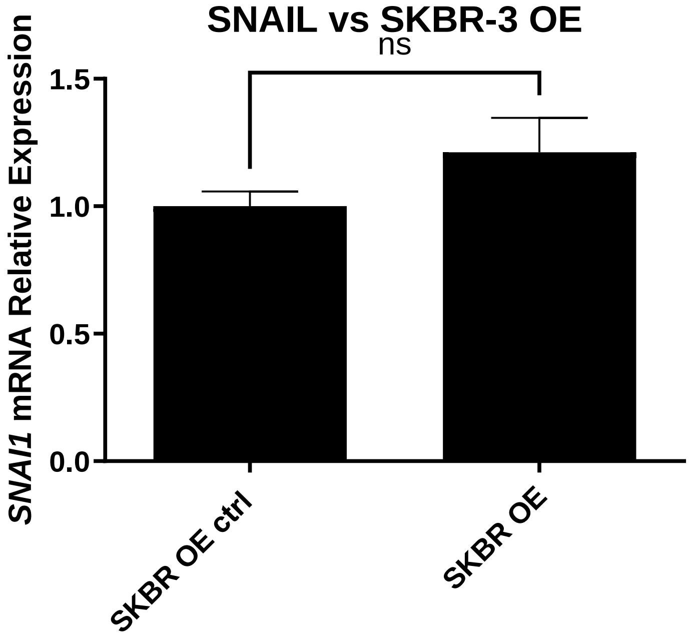

SNAIL vs SKBR-3 OE
SNAI1 mRNA Relative Expression
1.5
1.0
0.5
0.0
ns
SKBR OE ctrl
SKBR OE

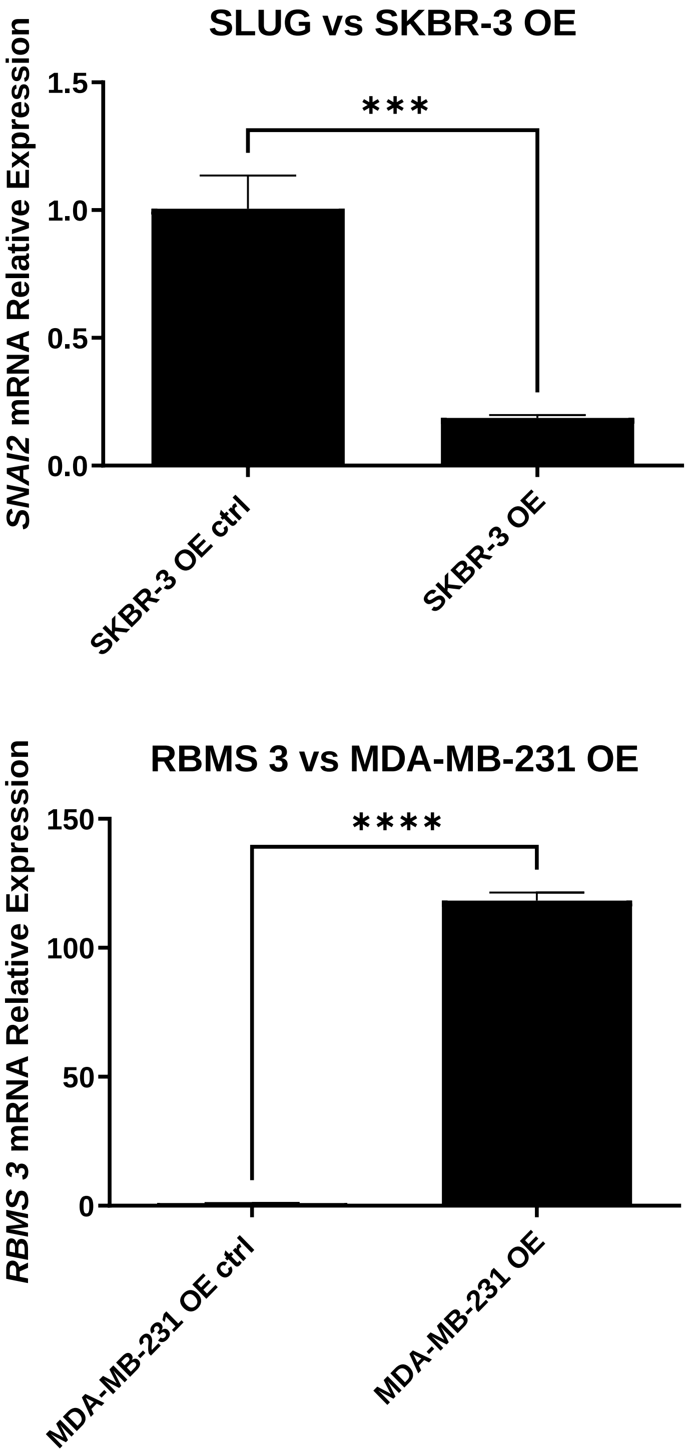

SLUG vs SKBR-3 OE
SNAI2 mRNA Relative Expression
1.5
1.0
0.5
0.0
***
SKBR-3 OE ctrl
SKBR-3 OE
RBMS 3 vs MDA-MB-231 OE
RBMS 3 mRNA Relative Expression
150
100
50
0
****
MDA-MB-231 OE ctrl
MDA-MB-231 OE

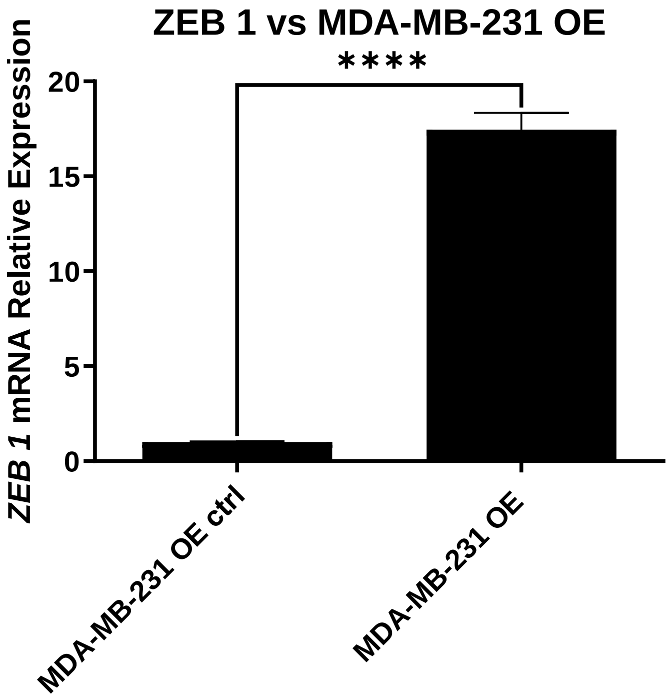

ZEB 1 vs MDA-MB-231 OE
****
ZEB 1 mRNA Relative Expression
20
15
10
5
0
MDA-MB-231 OE ctrl
MDA-MB-231 OE

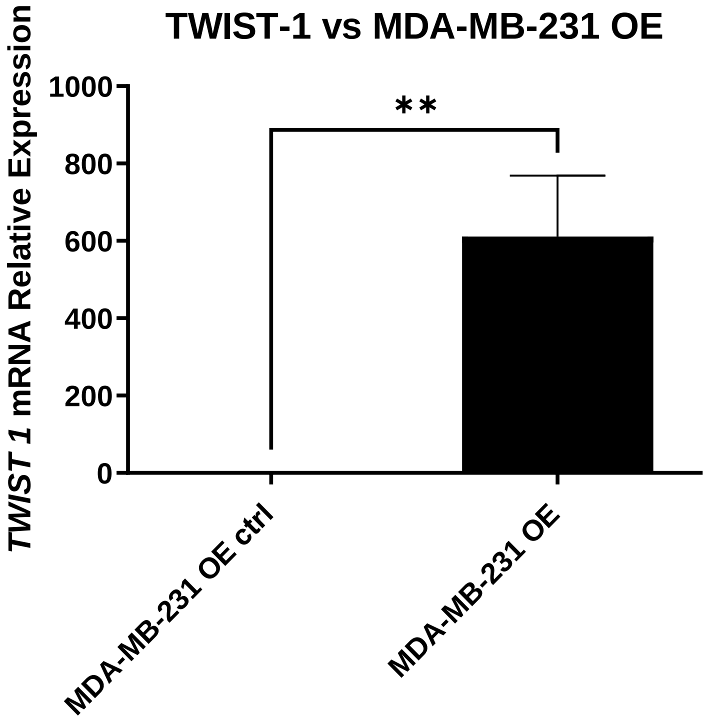

TWIST-1 vs MDA-MB-231 OE
**
TWIST 1 mRNA Relative Expression
1000
800
600
400
200
0
MDA-MB-231 OE ctrl
MDA-MB-231 OE

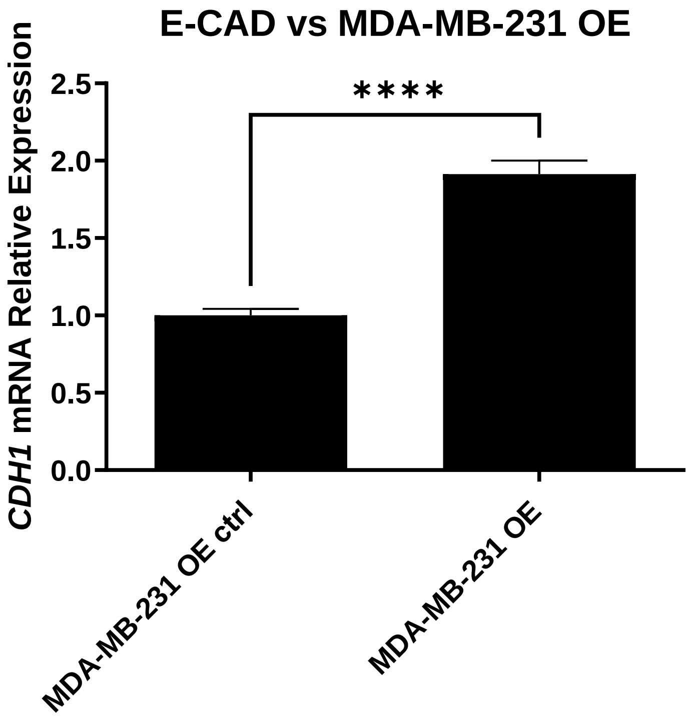
E-CAD vs MDA-MB-231 OE
CDH1 mRNA Relative Expression
2.5
2.0
1.5
1.0
0.5
0.0
****
MDA-MB-231 OE ctrl
MDA-MB-231 OE

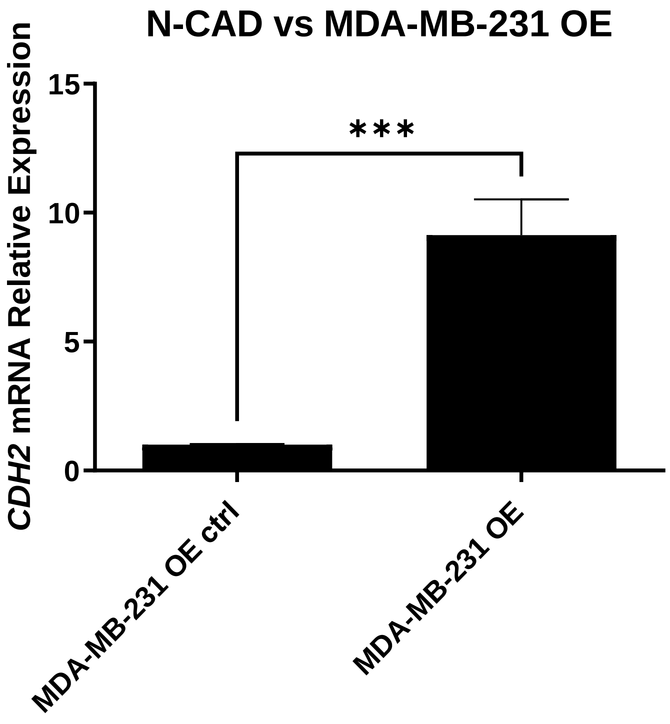
N-CAD vs MDA-MB-231 OE
CDH2 mRNA Relative Expression
15
10
5
0
***
MDA-MB-231 OE ctrl
MDA-MB-231 OE

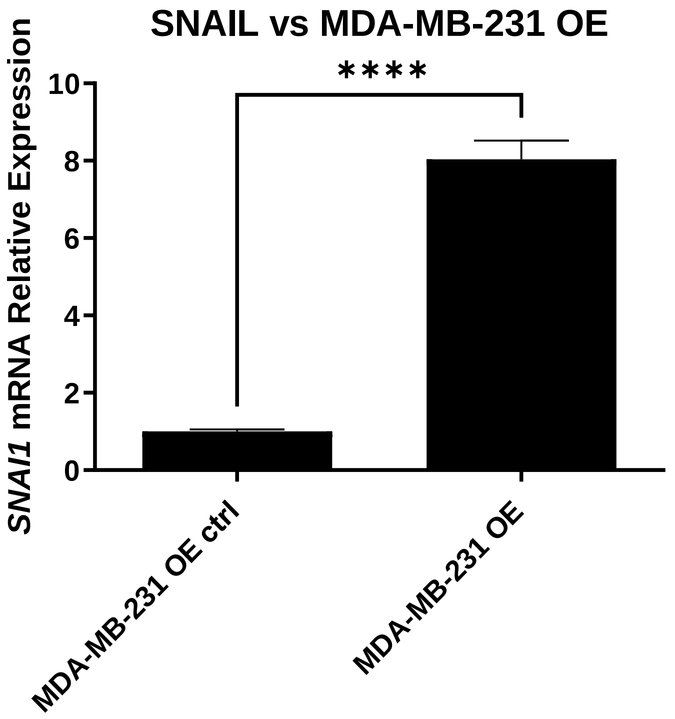

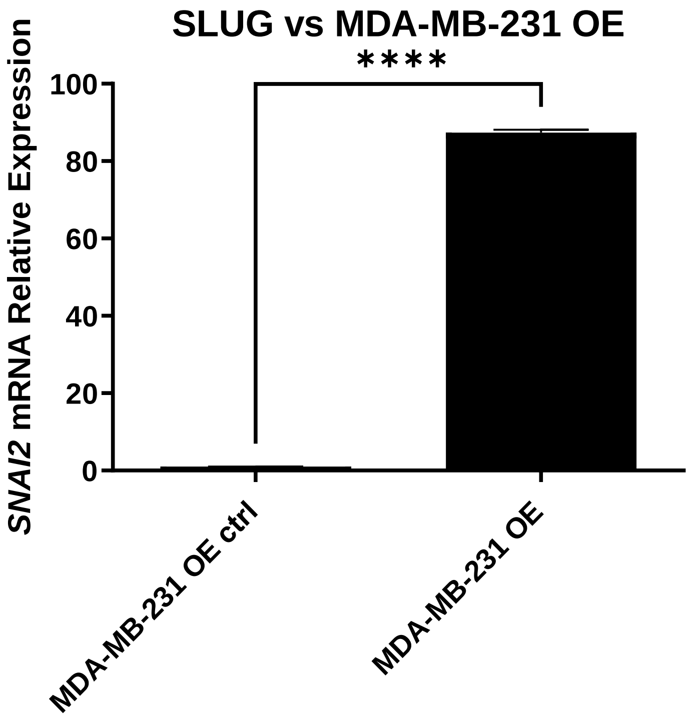

2. **RT-qPCR results for RBMS 3 silencing**

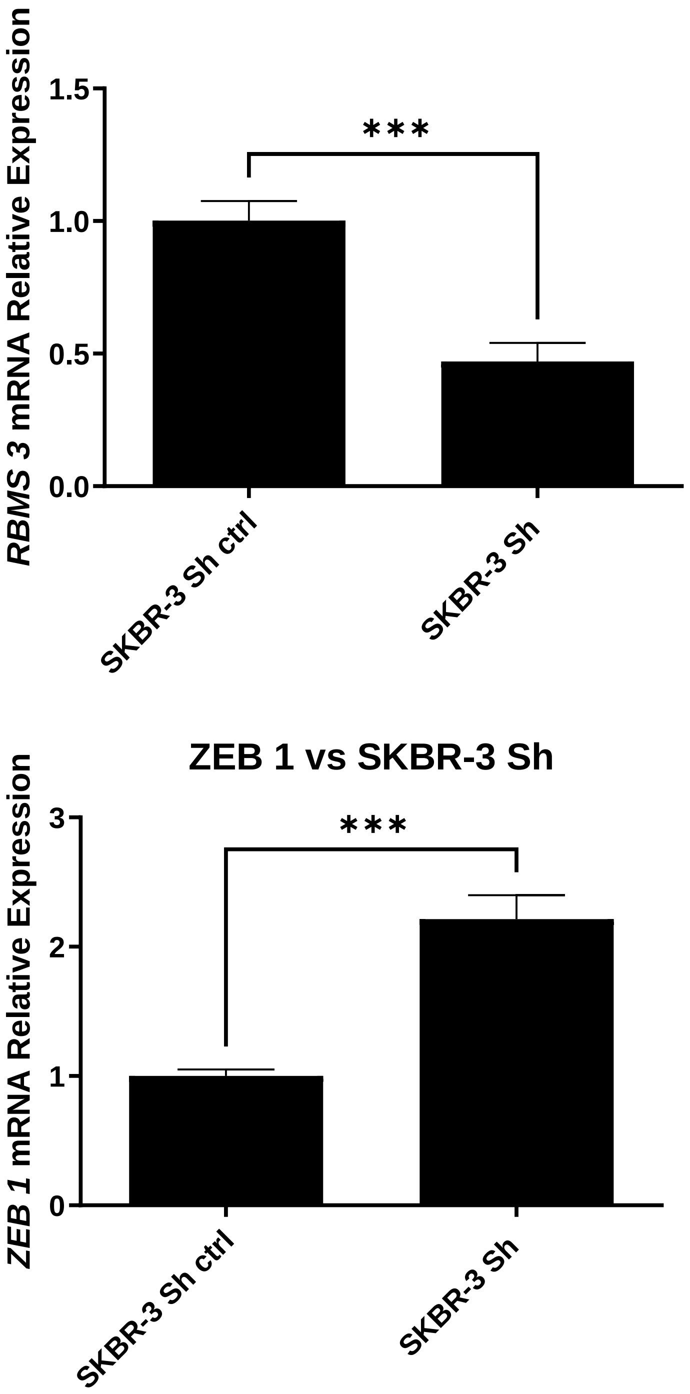

RBMS 3 mRNA Relative Expression
1.5
1.0
0.5
0.0
***
SKBR-3 Sh ctrl
SKBR-3 Sh
ZEB 1 vs SKBR-3 Sh
ZEB 1 mRNA Relative Expression
3
2
1
0
***
SKBR-3 Sh ctrl
SKBR-3 Sh

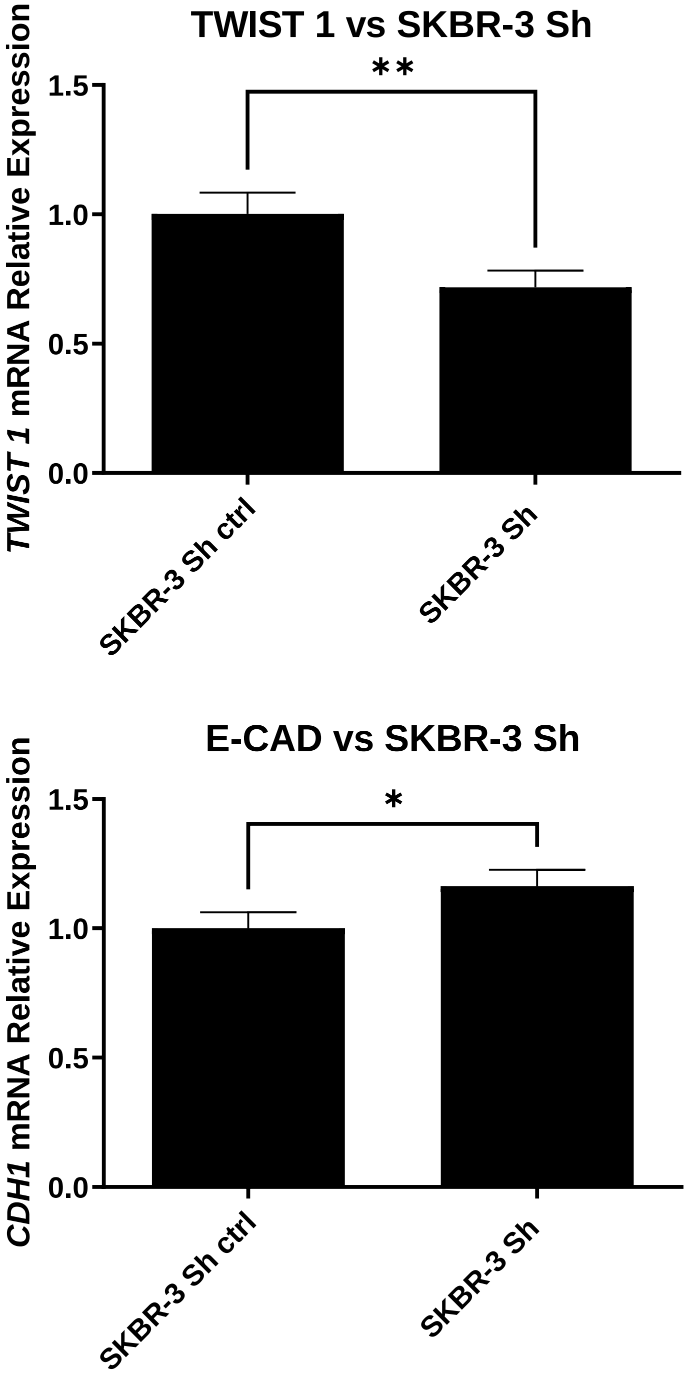
TWIST 1 vs SKBR-3 Sh
TWIST 1 mRNA Relative Expression
**
1.5
1.0
0.5
0.0
SKBR-3 Sh ctrl
SKBR-3 Sh
E-CAD vs SKBR-3 Sh
CDH1 mRNA Relative Expression
*
1.5
1.0
0.5
0.0
SKBR-3 Sh ctrl
SKBR-3 Sh

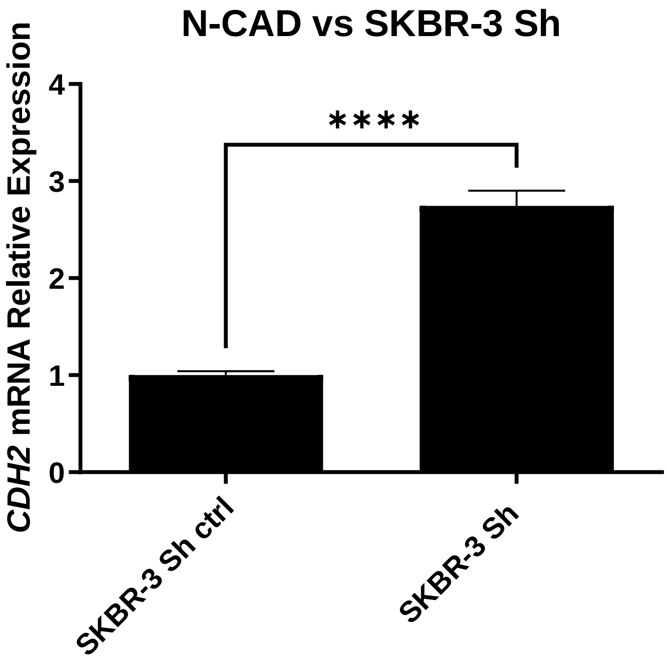
N-CAD vs SKBR-3 Sh
CDH2 mRNA Relative Expression
0
1
2
3
4
****
SKBR-3 Sh ctrl
SKBR-3 Sh

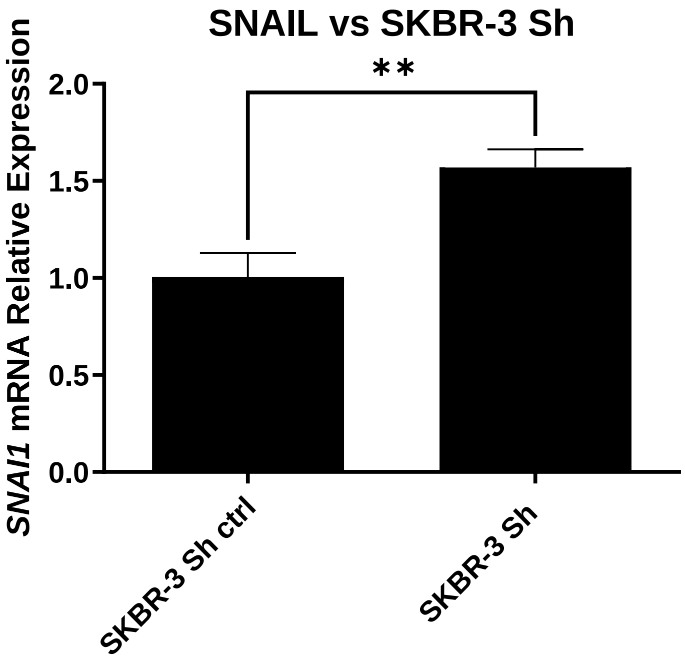
SNAIL vs SKBR-3 Sh
SNAI1 mRNA Relative Expression
0.0
0.5
1.0
1.5
2.0
**
SKBR-3 Sh ctrl
SKBR-3 Sh

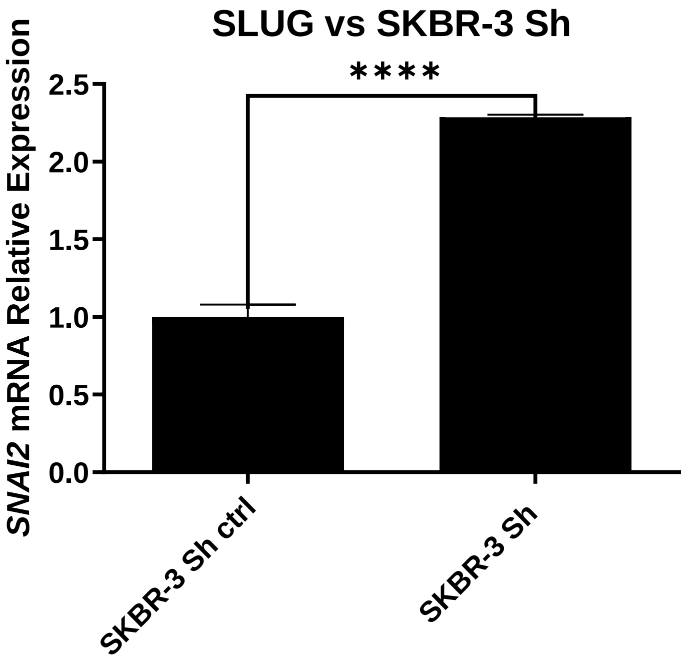
SLUG vs SKBR-3 Sh
SNAI2 mRNA Relative Expression
2.5
2.0
1.5
1.0
0.5
0.0
****
SKBR-3 Sh ctrl
SKBR-3 Sh

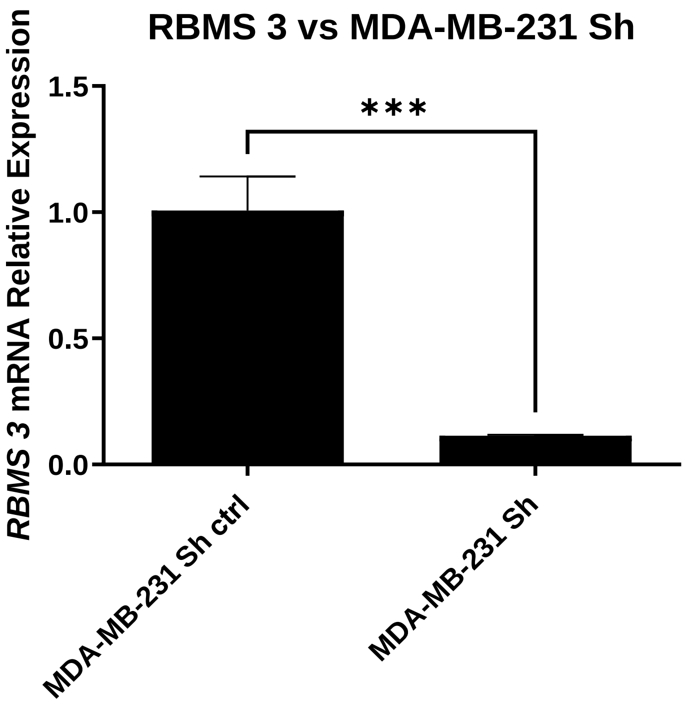
RBMS 3 vs MDA-MB-231 Sh
RBMS 3 mRNA Relative Expression
1.5
1.0
0.5
0.0
***
MDA-MB-231 Sh ctrl
MDA-MB-231 Sh

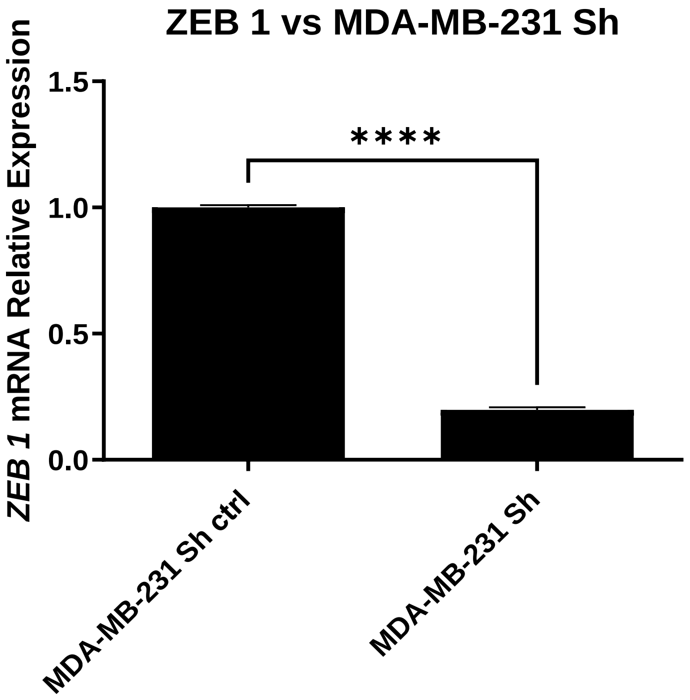
ZEB 1 vs MDA-MB-231 Sh
ZEB 1 mRNA Relative Expression
1.5
1.0
0.5
0.0
****
MDA-MB-231 Sh ctrl
MDA-MB-231 Sh

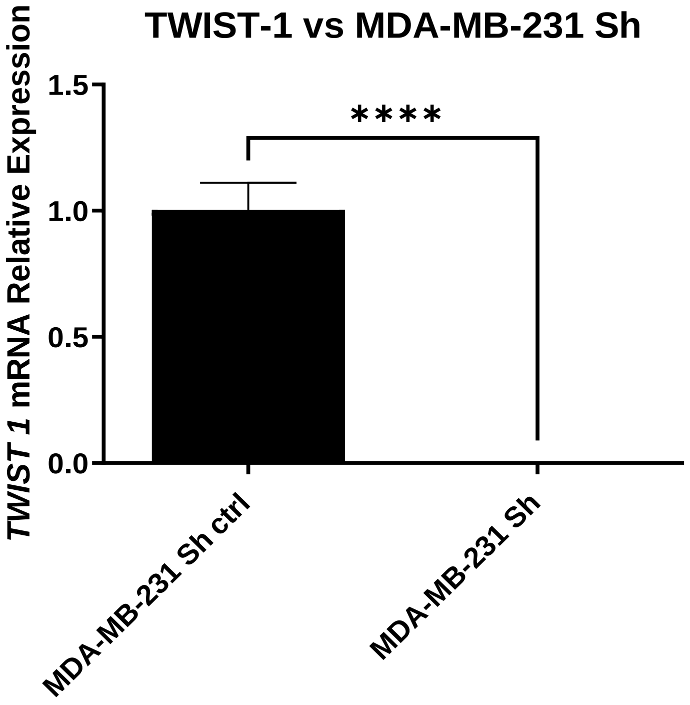
TWIST-1 vs MDA-MB-231 Sh
TWIST 1 mRNA Relative Expression
1.5
1.0
0.5
0.0
****
MDA-MB-231 Sh ctrl
MDA-MB-231 Sh

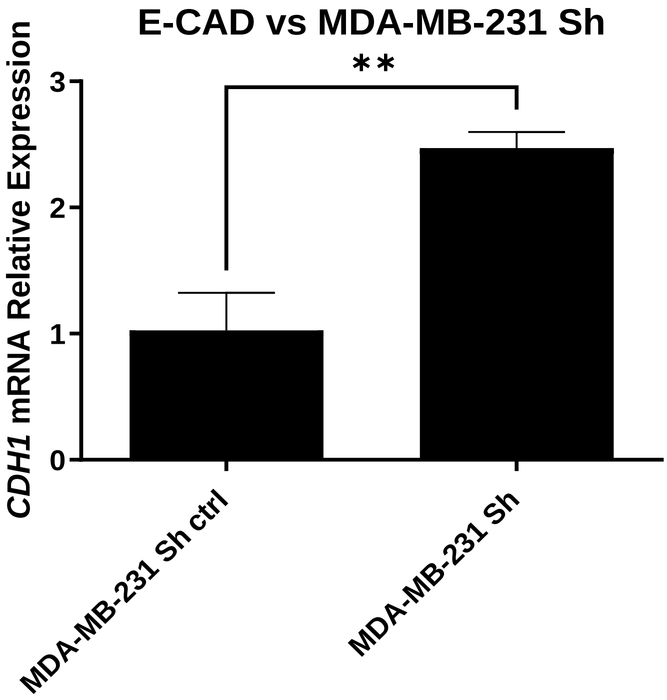
E-CAD vs MDA-MB-231 Sh
**
CDH1 mRNA Relative Expression
0
1
2
3
MDA-MB-231 Sh ctrl
MDA-MB-231 Sh

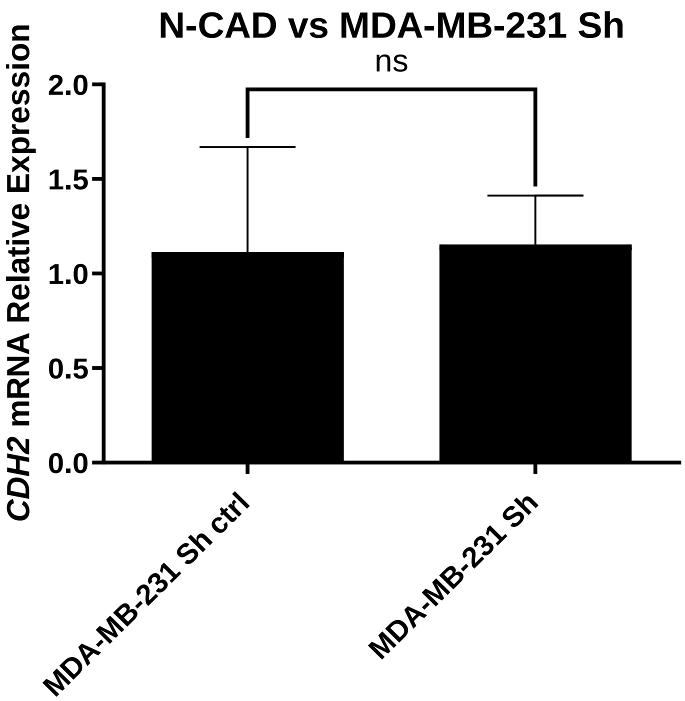
N-CAD vs MDA-MB-231 Sh
ns
CDH2 mRNA Relative Expression
0.0
0.5
1.0
1.5
2.0
MDA-MB-231 Sh ctrl
MDA-MB-231 Sh

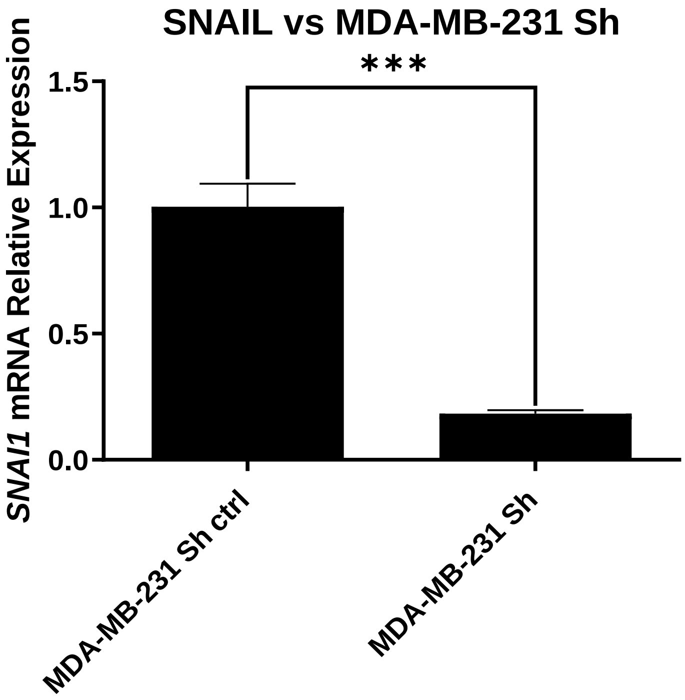
SNAIL vs MDA-MB-231 Sh
***
SNAI1 mRNA Relative Expression
1.5
1.0
0.5
0.0
MDA-MB-231 Sh ctrl
MDA-MB-231 Sh

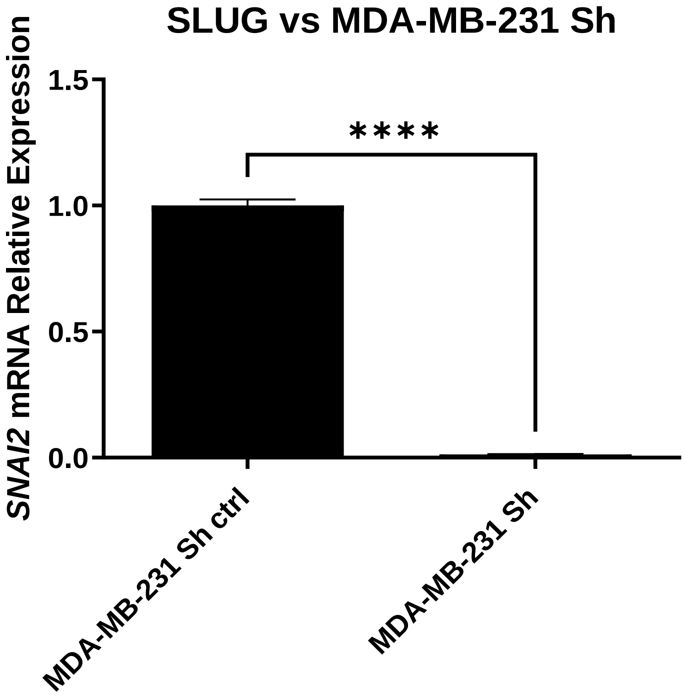
SLUG vs MDA-MB-231 Sh
****
SNAI2 mRNA Relative Expression
1.5
1.0
0.5
0.0
MDA-MB-231 Sh ctrl
MDA-MB-231 Sh

Original Blot Images

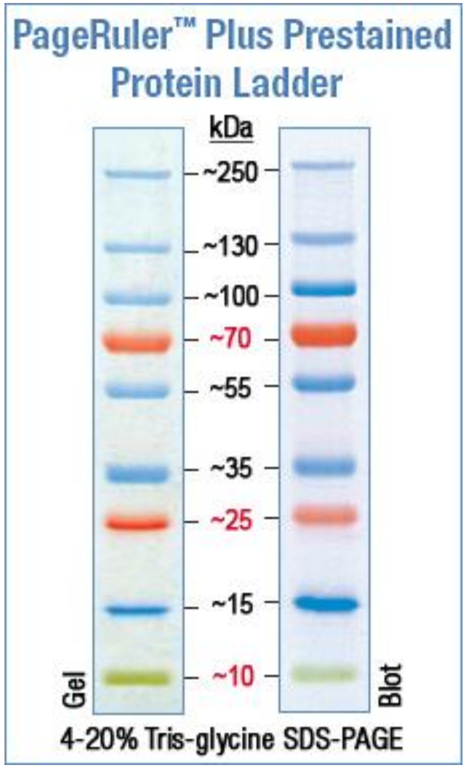

Protein Ladder used in all Blots.

1. **RBMS 3 – 55kDa**
   a) **MDA-MB-231**

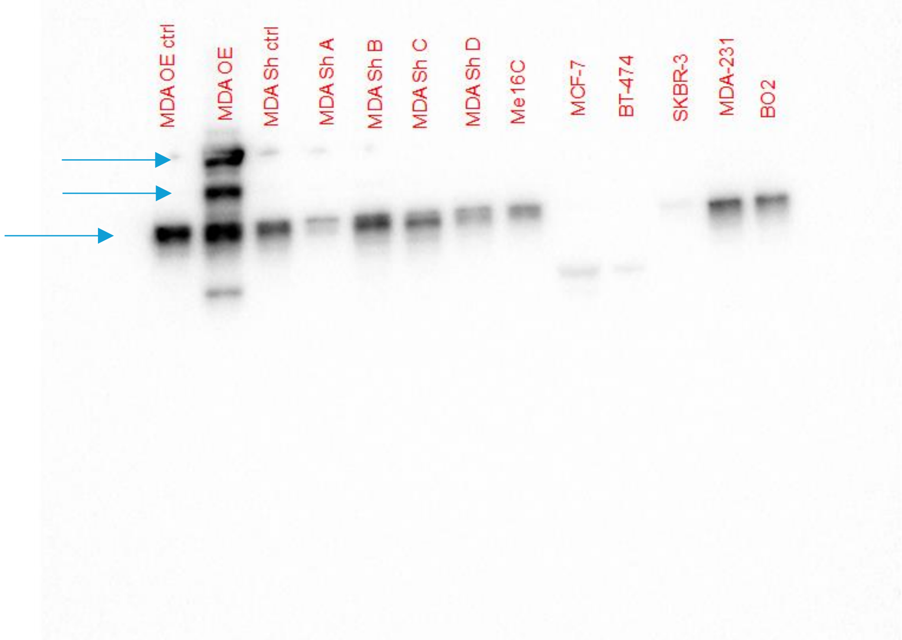

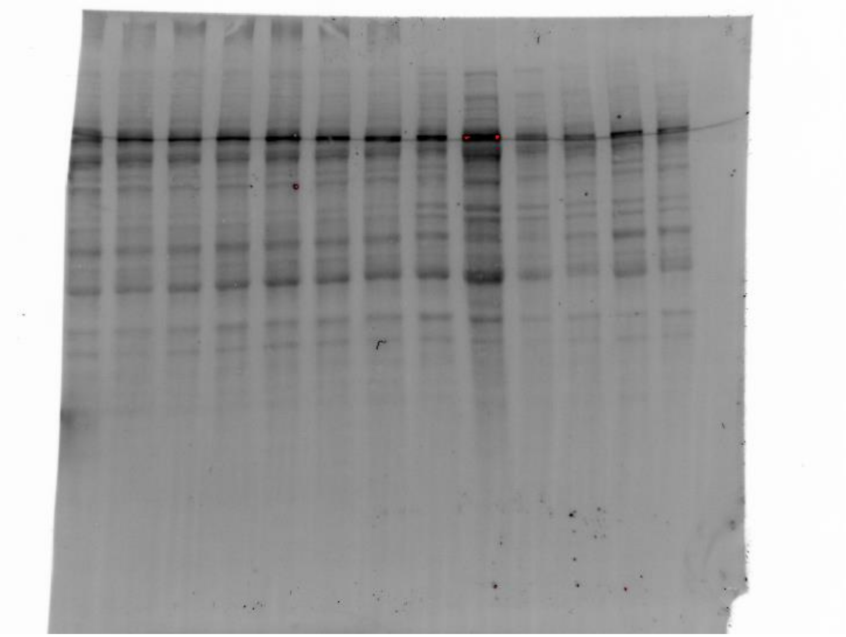

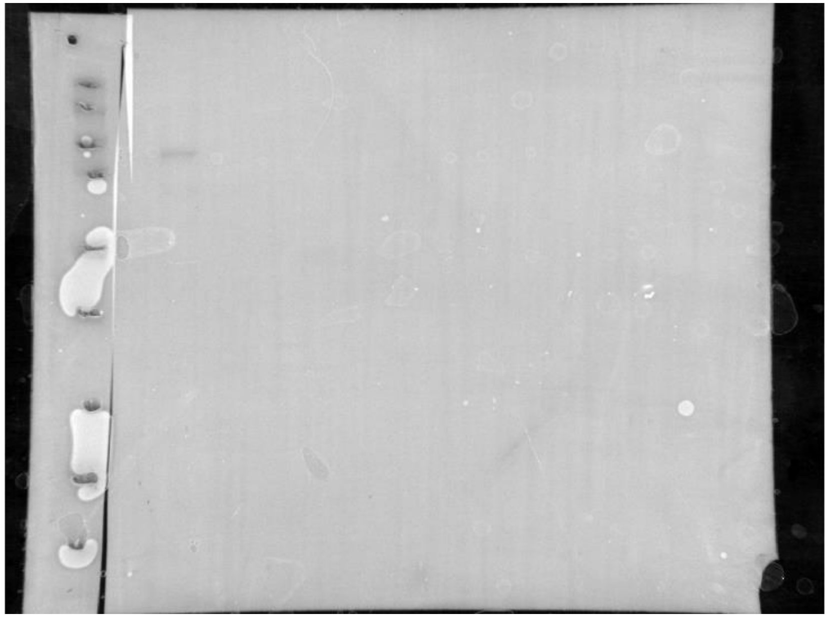

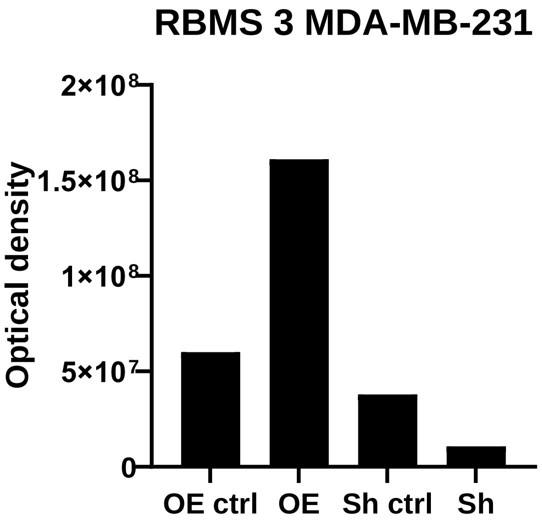

b) SKBR-3

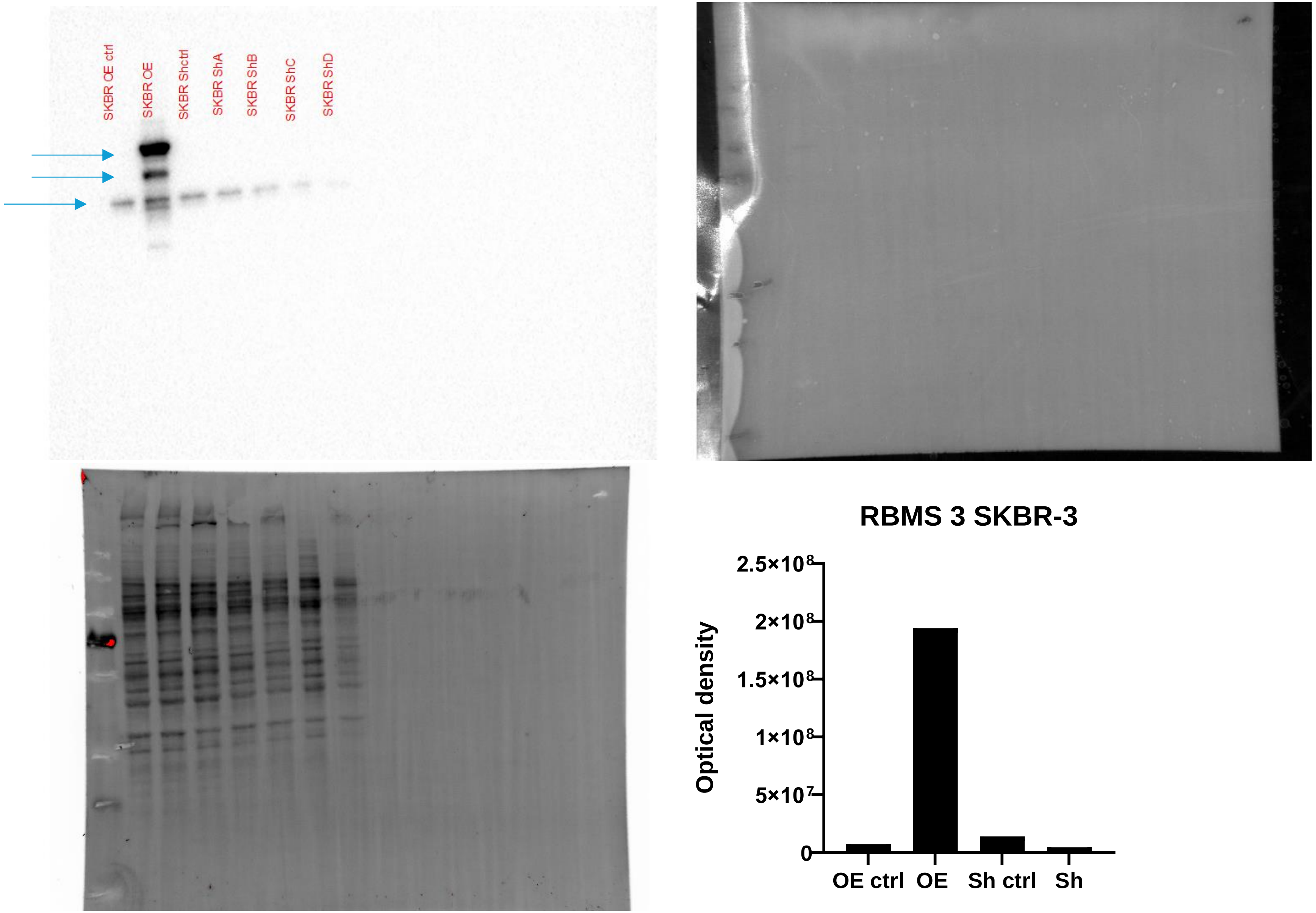
SKBR OE ctrl
SKBR OE
SKBR Shctrl
SKBR ShA
SKBR ShB
SKBR ShC
SKBR ShD
RBMS 3 SKBR-3
Optical density
2.5×10^8
2×10^8
1.5×10^8
1×10^8
5×10^7
0
OE ctrl
OE
Sh ctrl
Sh

2. **E-CAD- 110kDa**

   a) **MDA-MB-231**

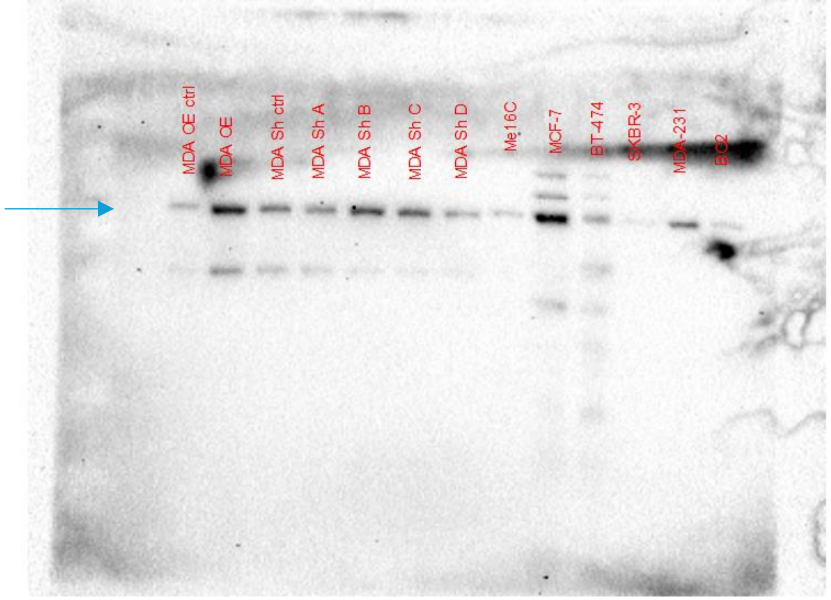

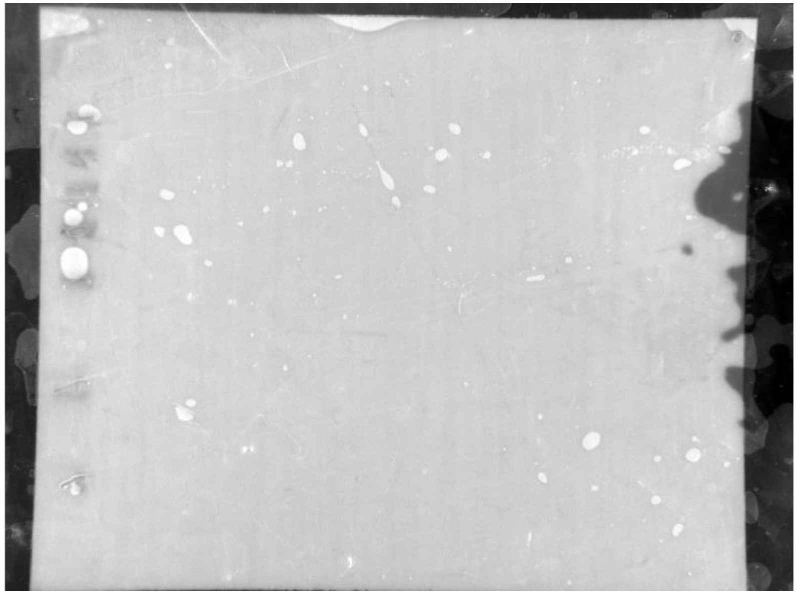

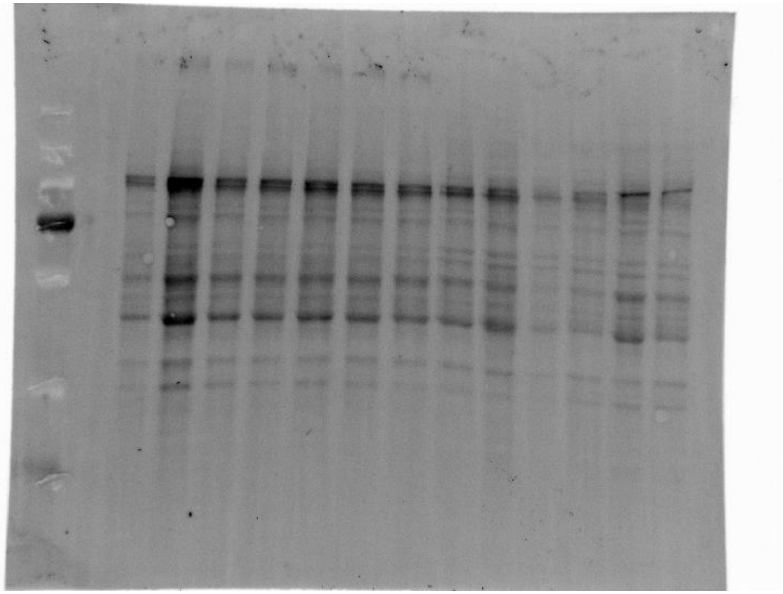

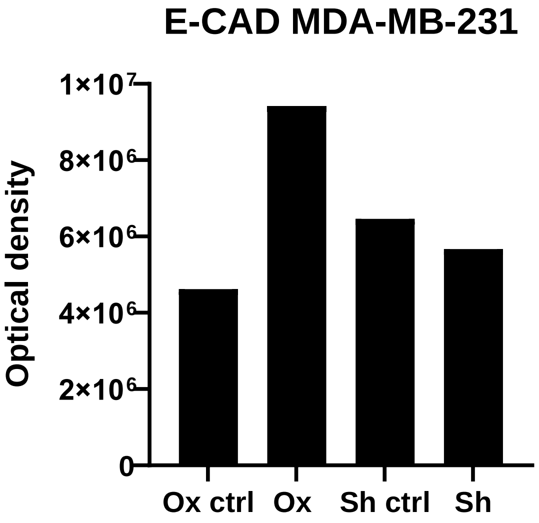

b) SKBR-3

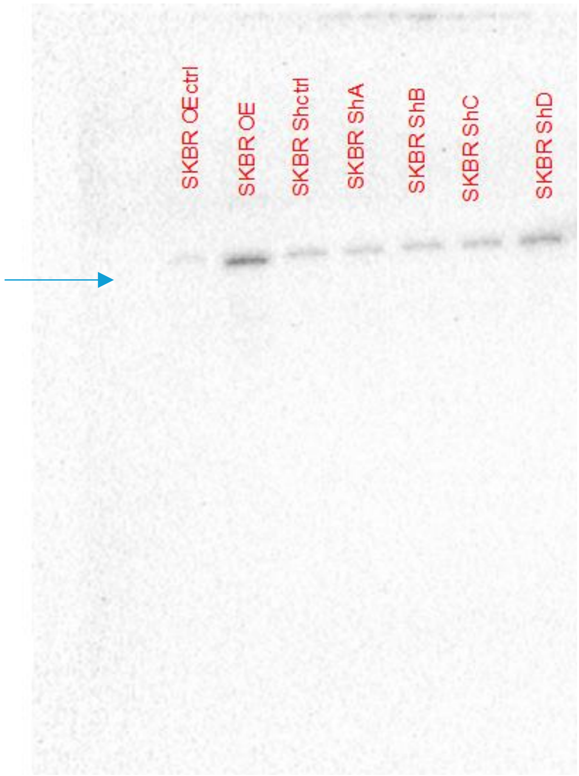

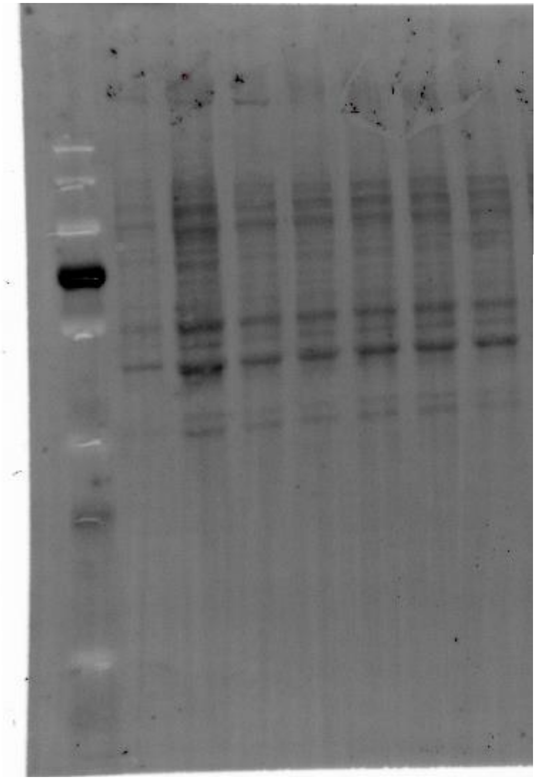

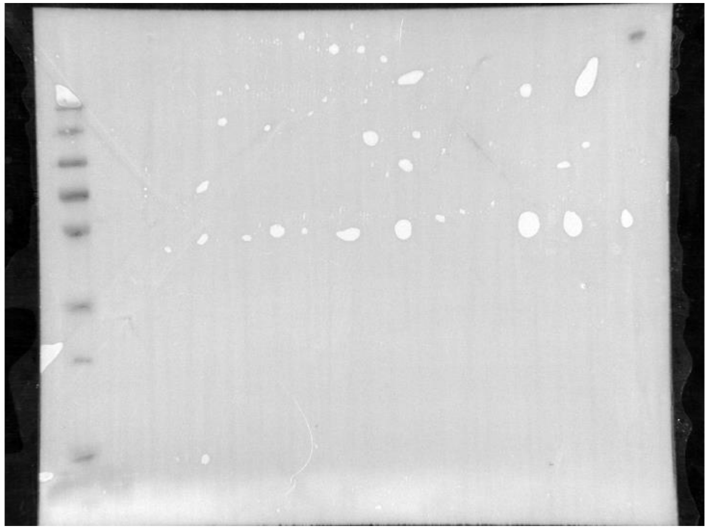

E-CAD SKBR-3

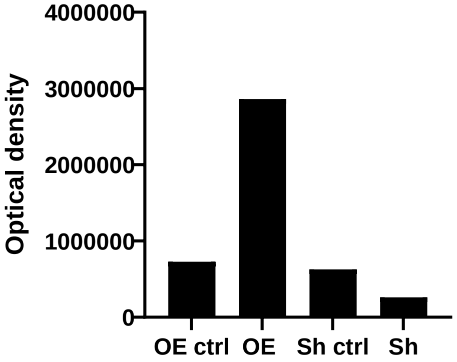

3. **N-CAD- 110kDa**
   a) **MDA-MB-231**

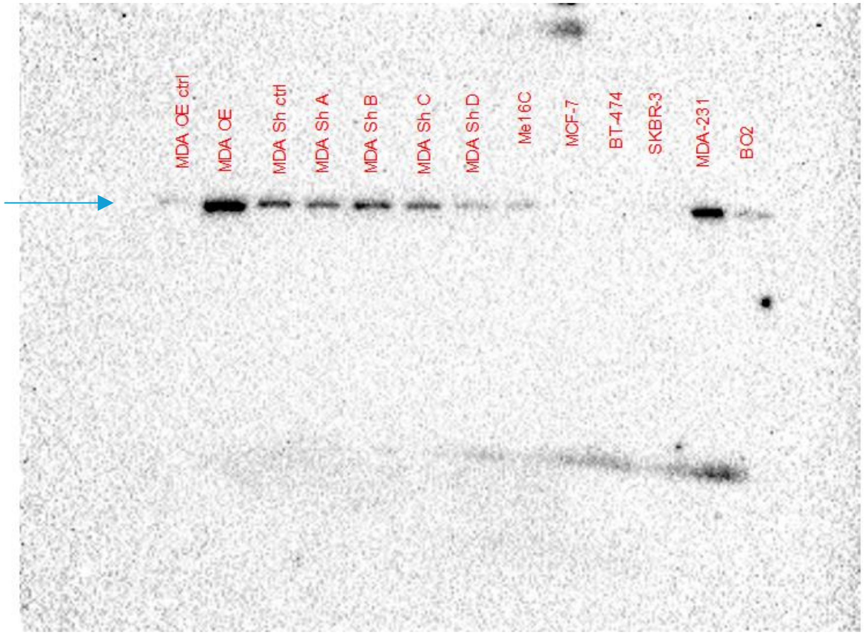

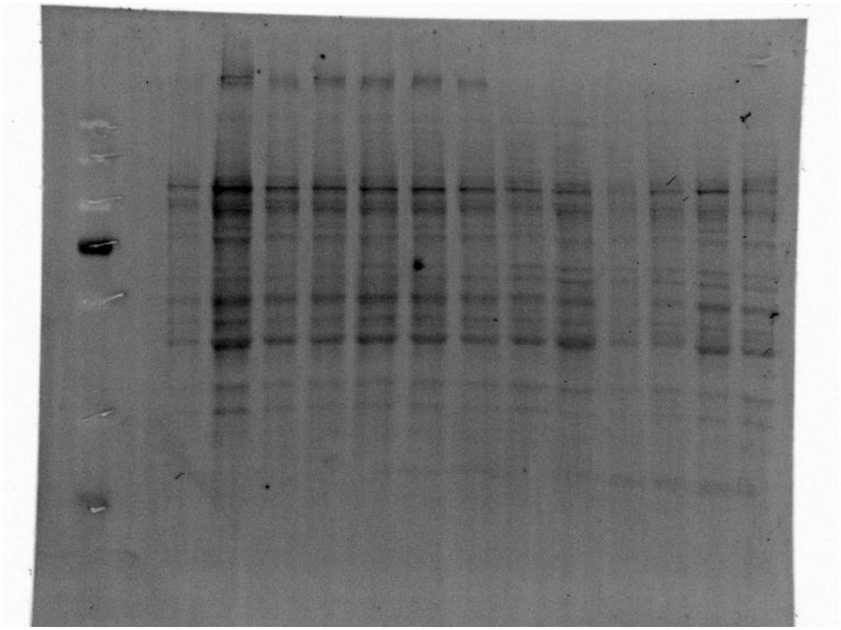

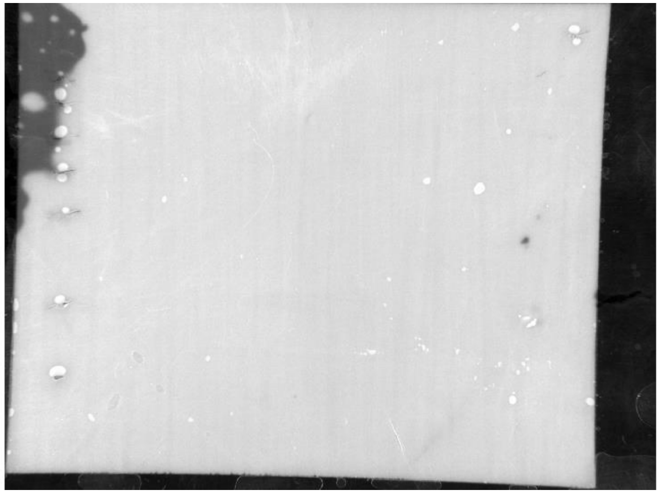

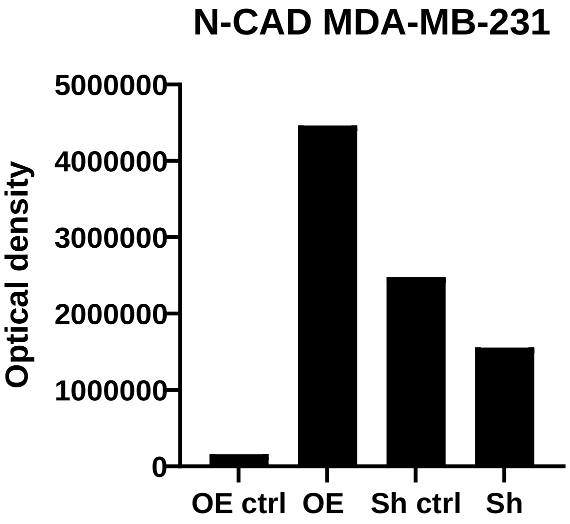

b) SKBR-3

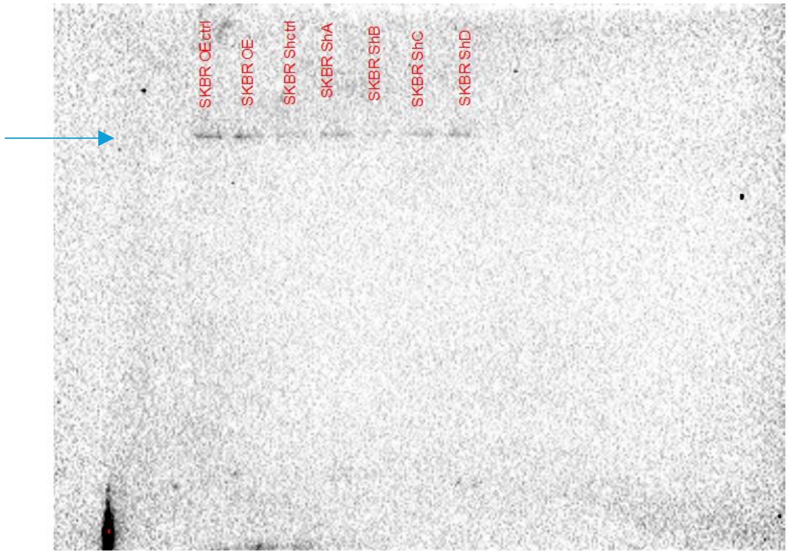
SKBR OEctrl
SKBR OE
SKBR Shctrl
SKBR ShA
SKBR ShB
SKBR ShC
SKBR ShD

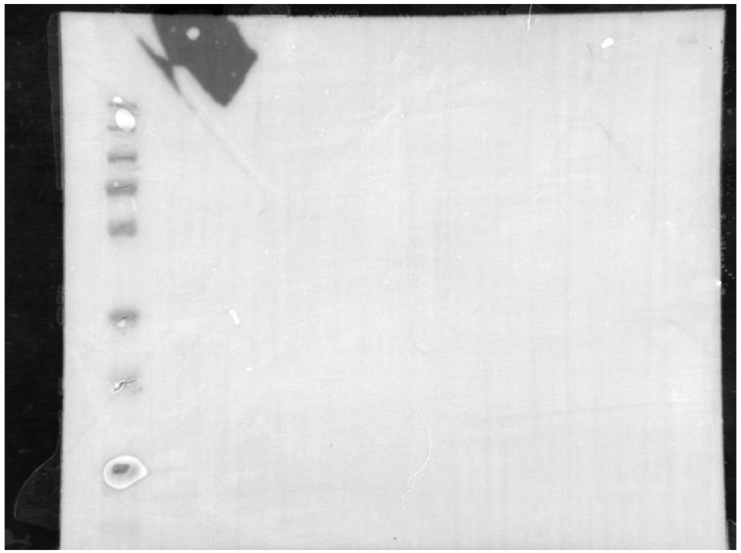

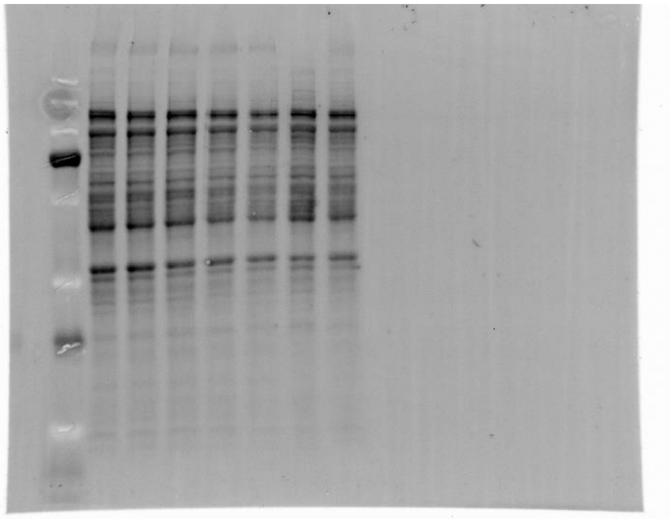

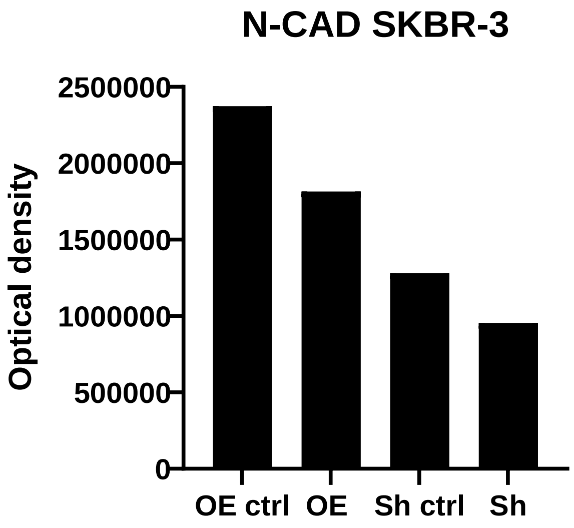
N-CAD SKBR-3
Optical density
2500000
2000000
1500000
1000000
500000
0
OE ctrl
OE
Sh ctrl
Sh

4. **SNAIL- 30kDa**

a) **MDA-MB-231**

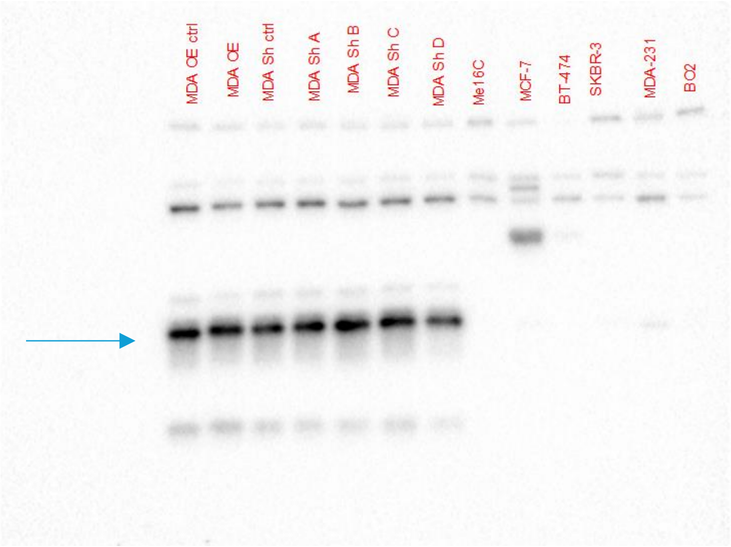

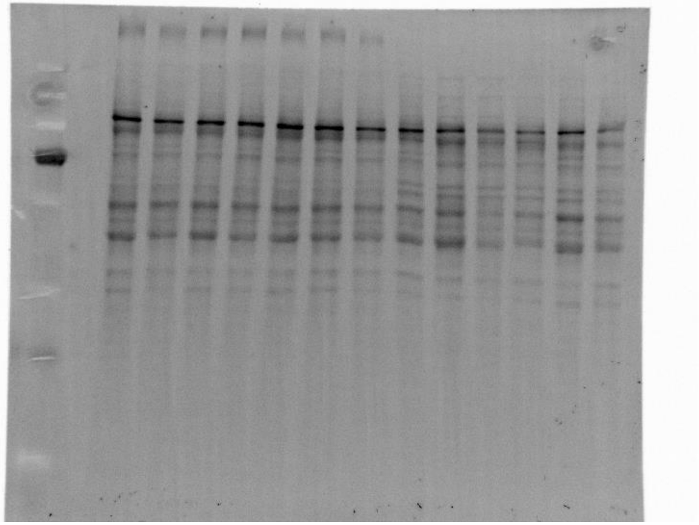

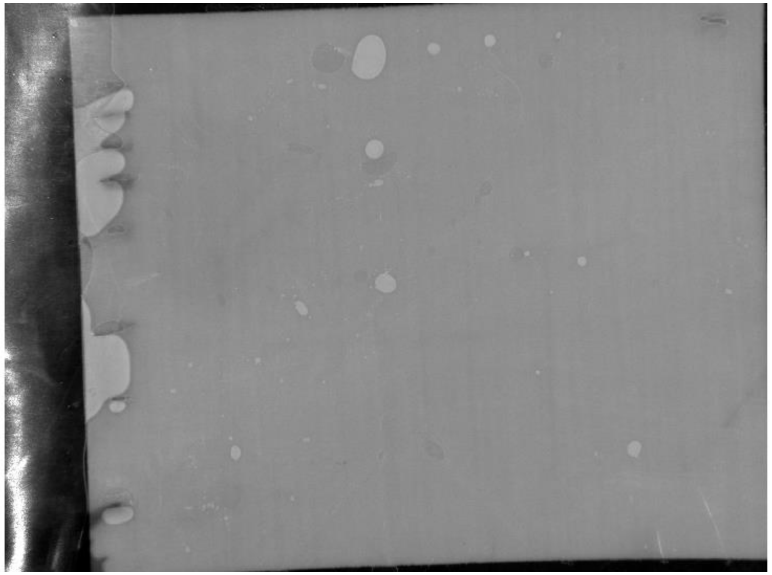

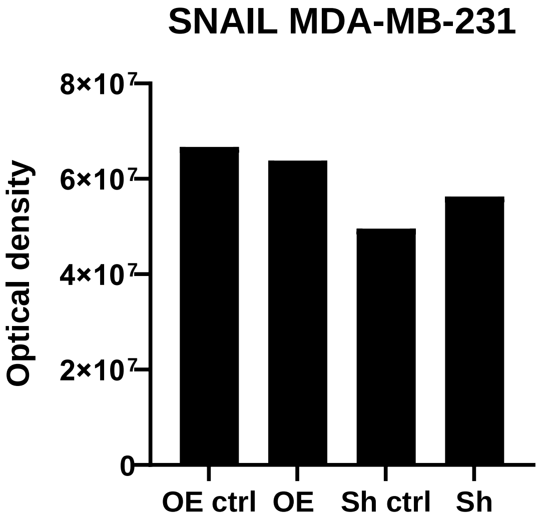

b) SKBR-3

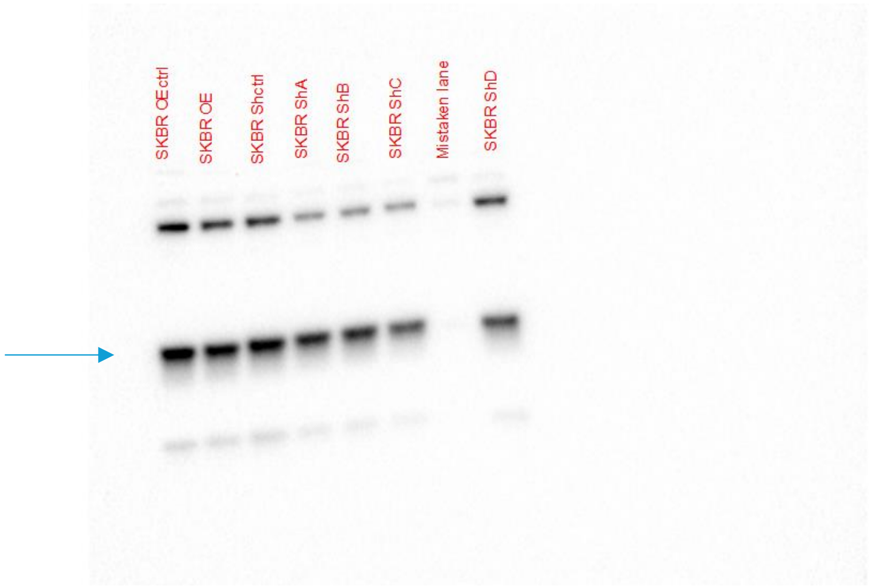
SKBR OEctrl
SKBR OE
SKBR Shctrl
SKBR ShA
SKBR ShB
SKBR ShC
Mistaken lane
SKBR ShD

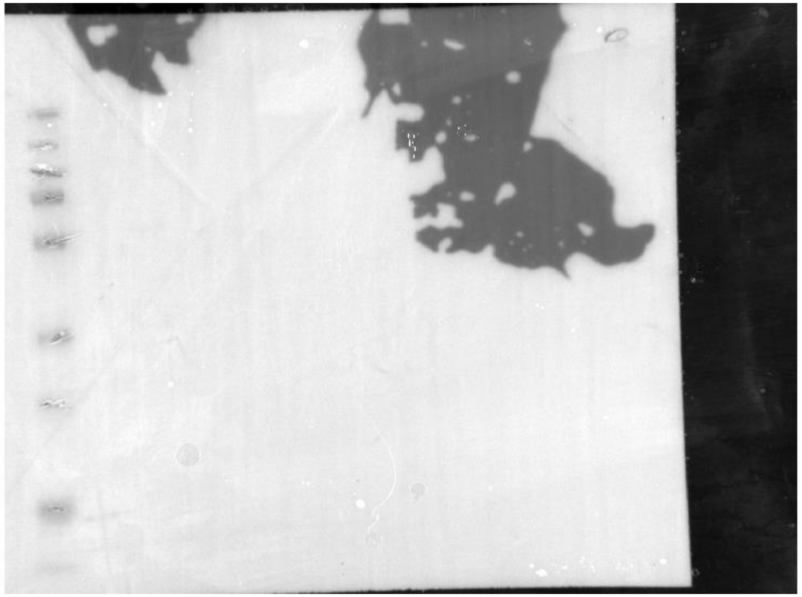

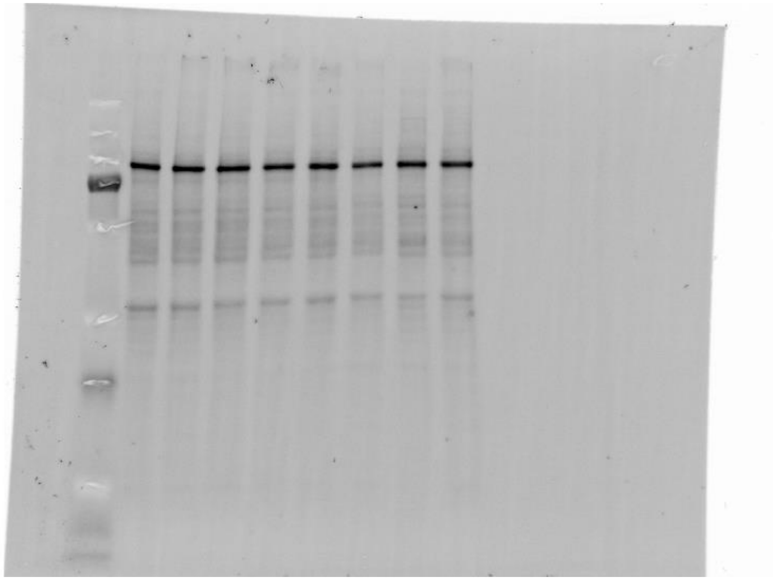

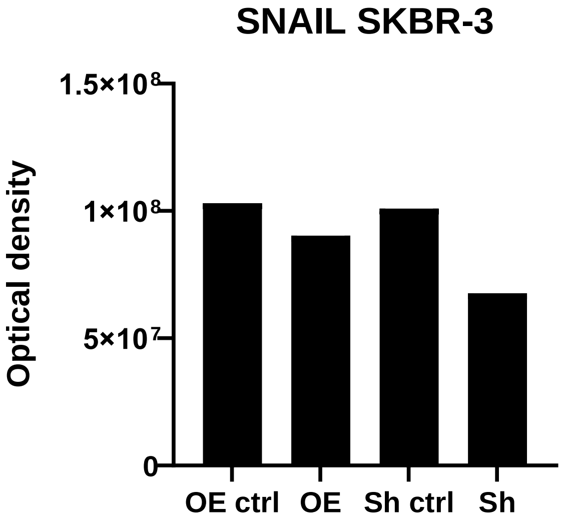
SNAIL SKBR-3
Optical density
1.5×10⁸
1×10⁸
5×10⁷
0
OE ctrl
OE
Sh ctrl
Sh

5. SLUG- 45 kDa

a) MDA-MB-231

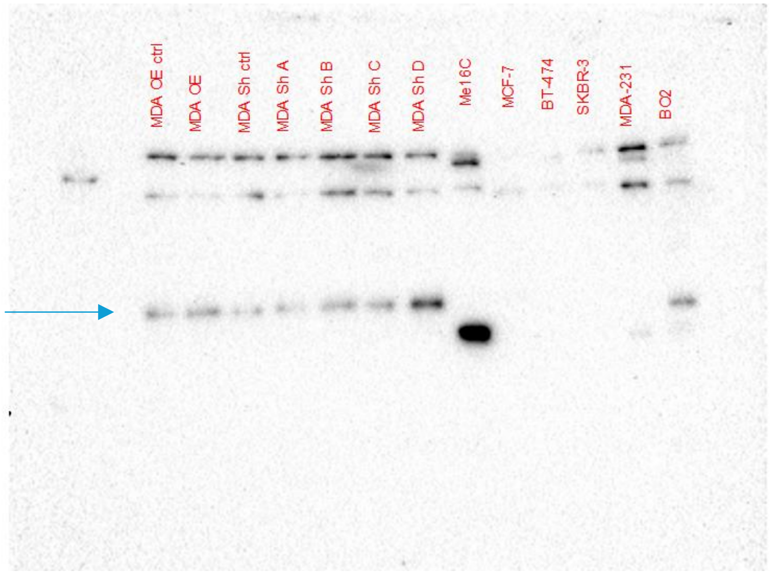

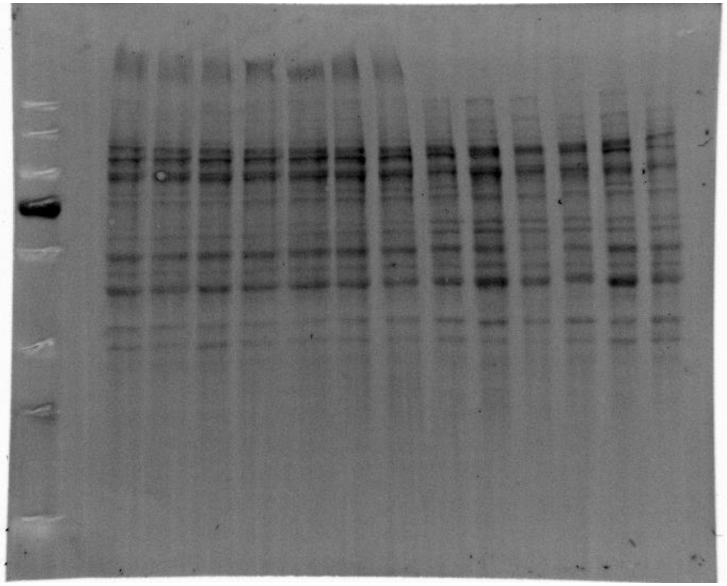

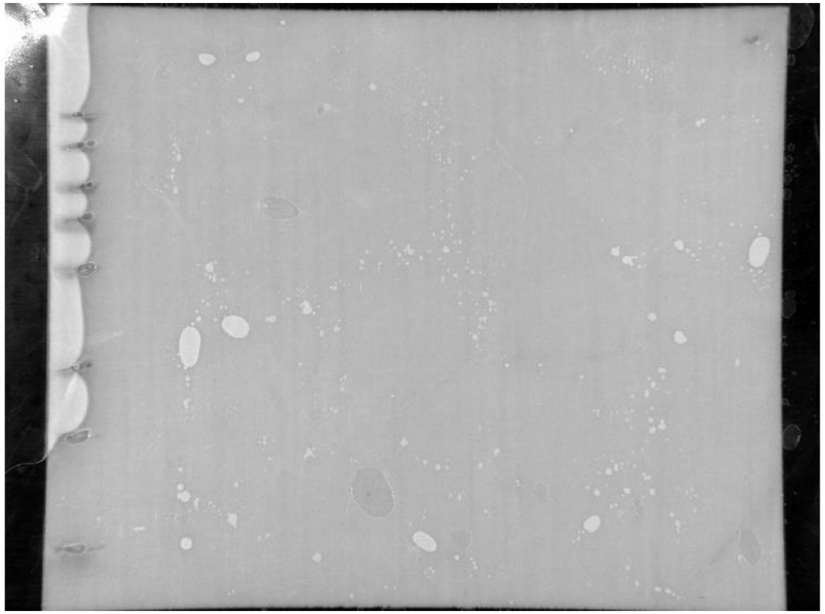

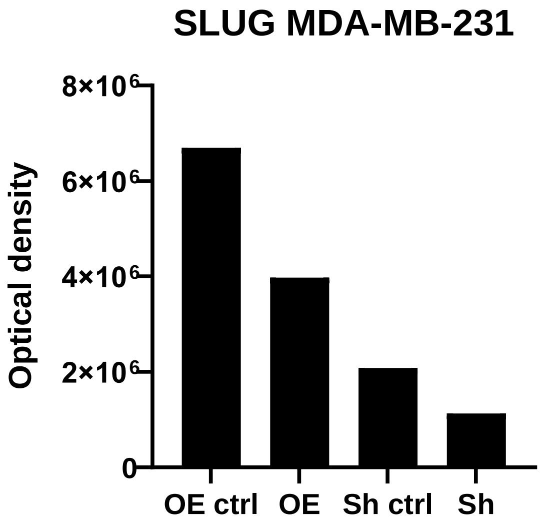

b) SKBR-3

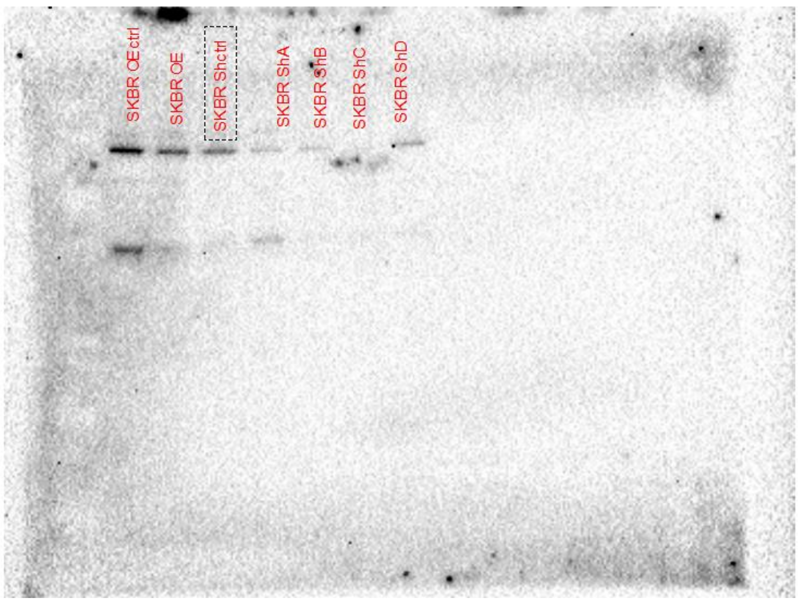
SKBR OEctrl
SKBR OE
SKBR Shctrl
SKBR ShA
SKBR ShB
SKBR ShC
SKBR ShD

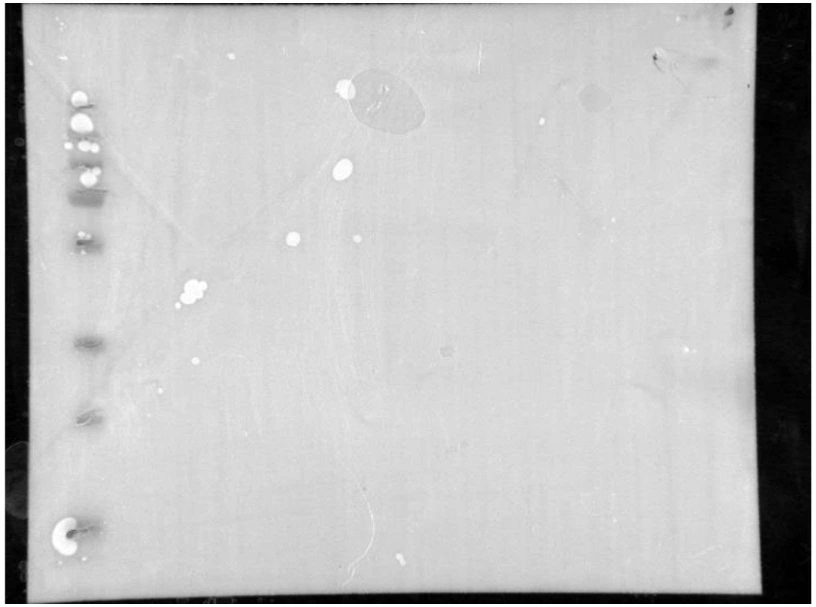

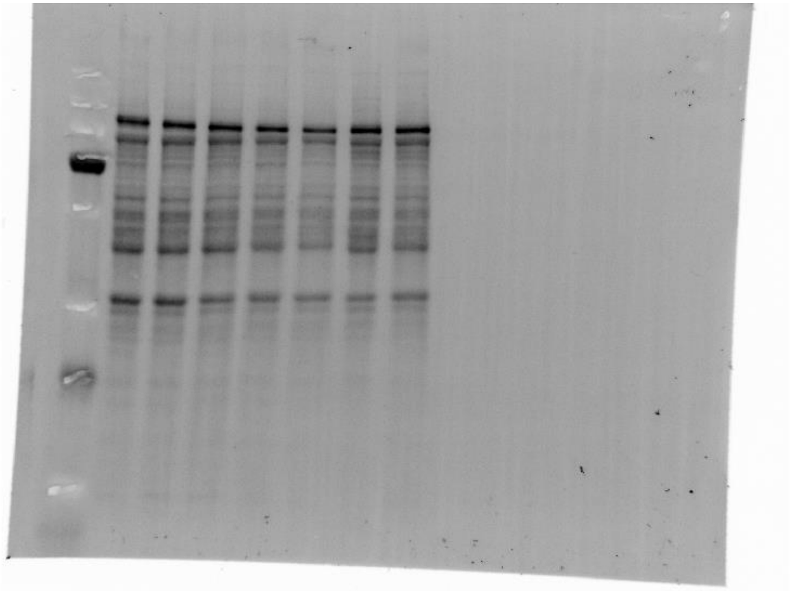

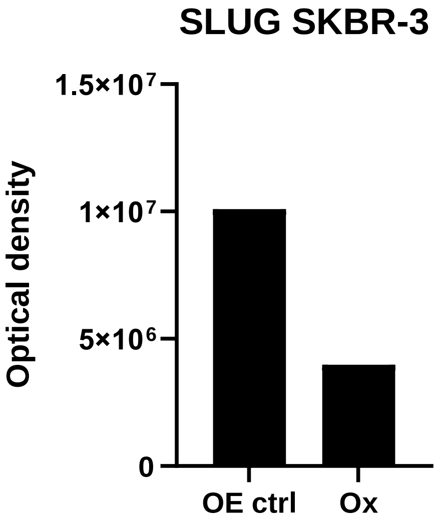
SLUG SKBR-3
Optical density
1.5×10^7
1×10^7
5×10^6
0
OE ctrl
Ox

6. **TWIST 1- 35kDa**

a) **MDA-MB-231**

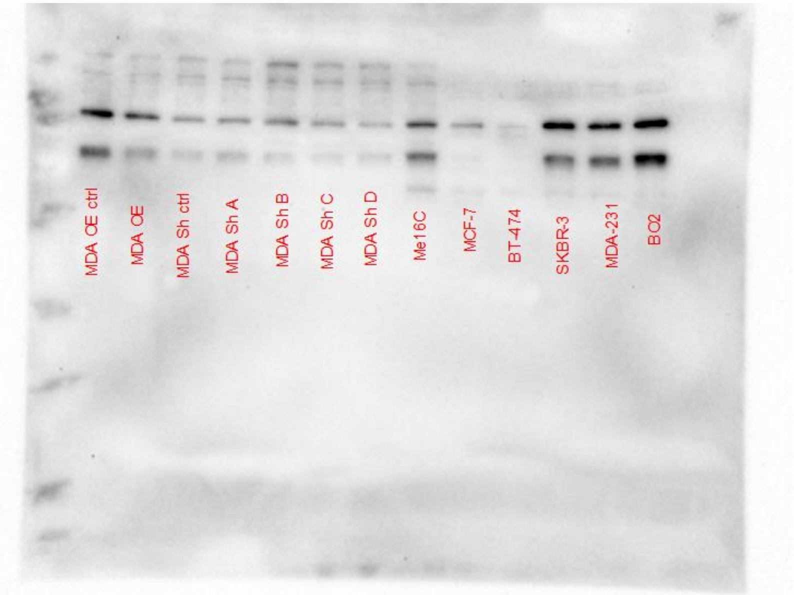

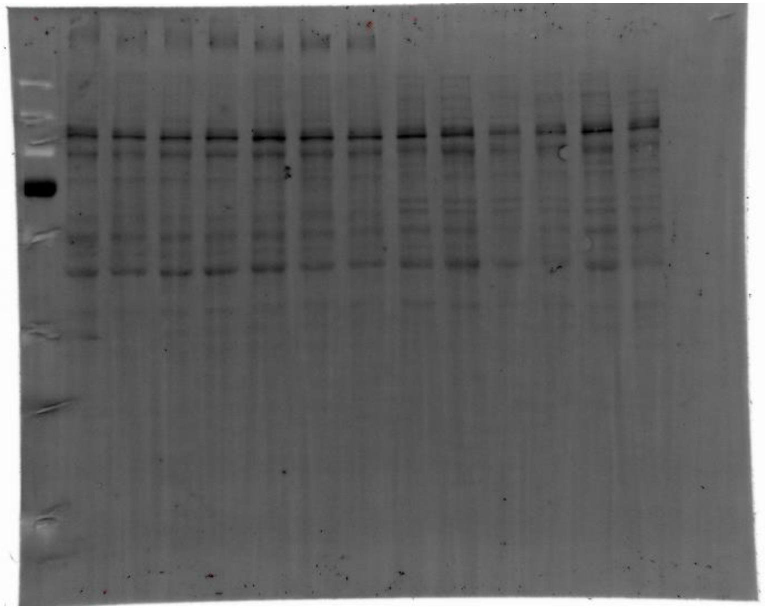

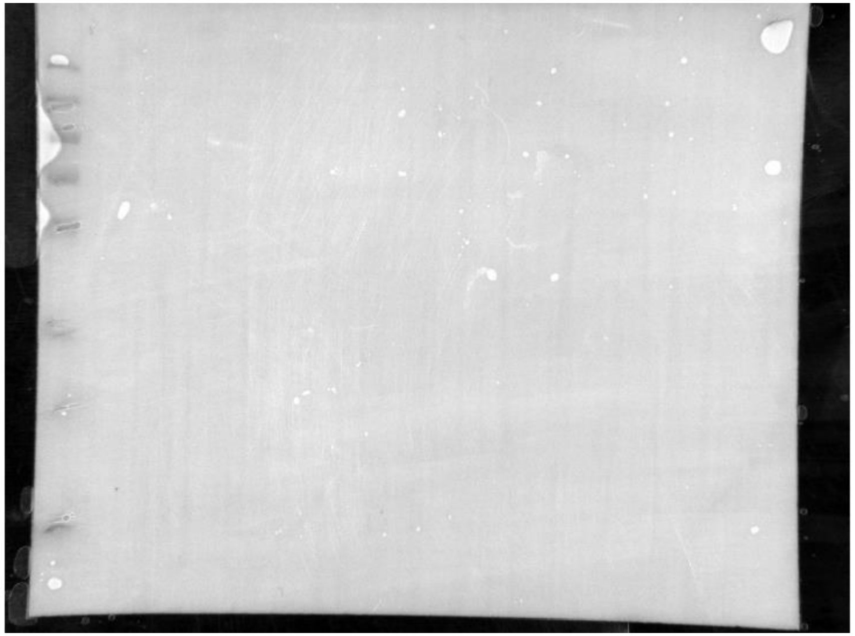

b) SKBR-3

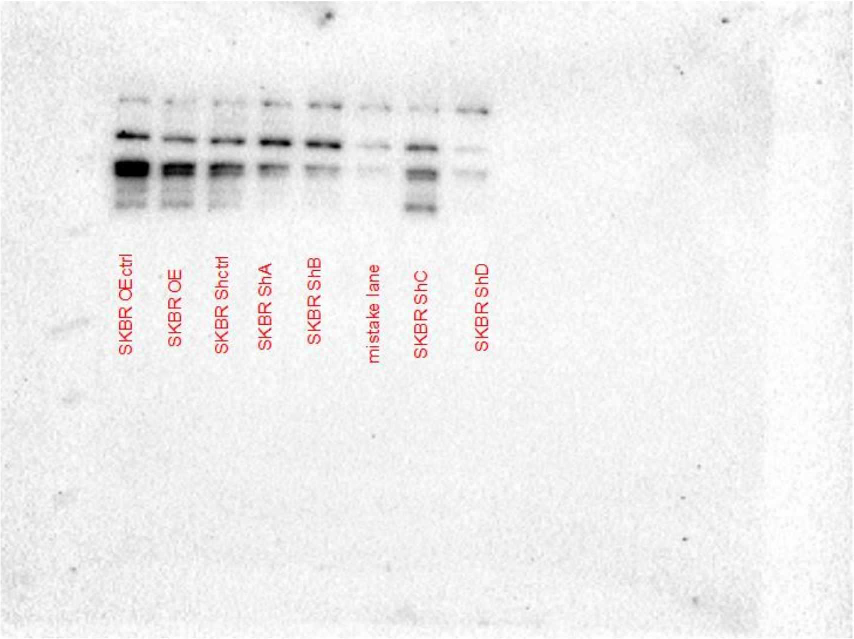

SKBR OEctrl
SKBR OE
SKBR Shctrl
SKBR ShA
SKBR ShB
mistake lane
SKBR ShC
SKBR ShD

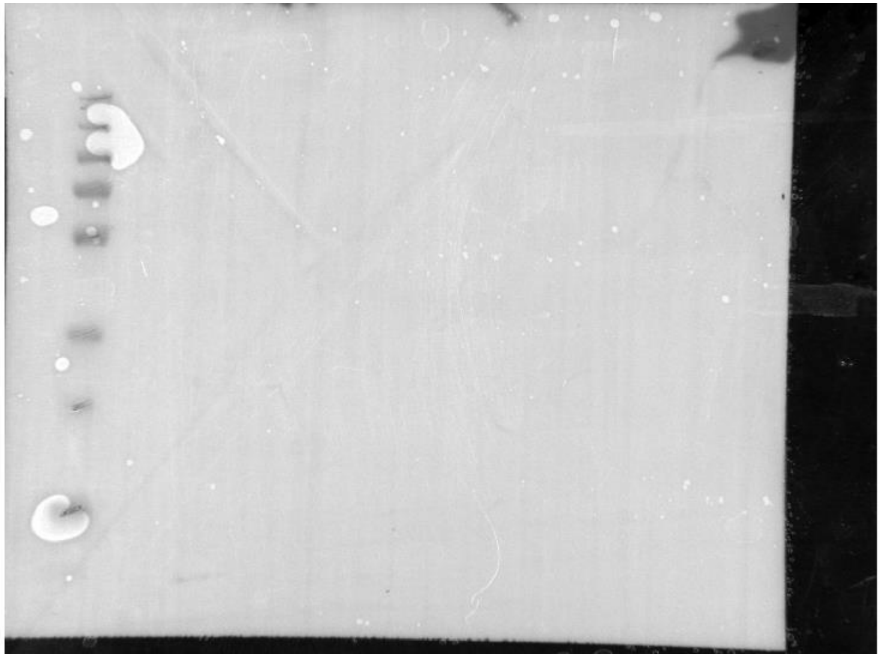

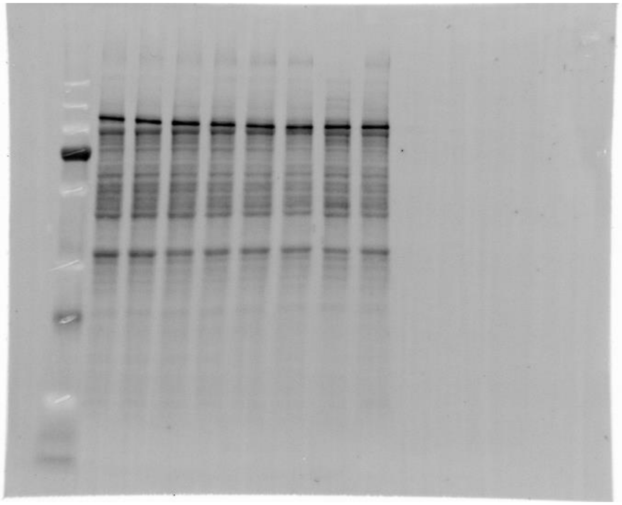

7. **ZEB 1- 220kDa**

a) **MDA-MB-231**

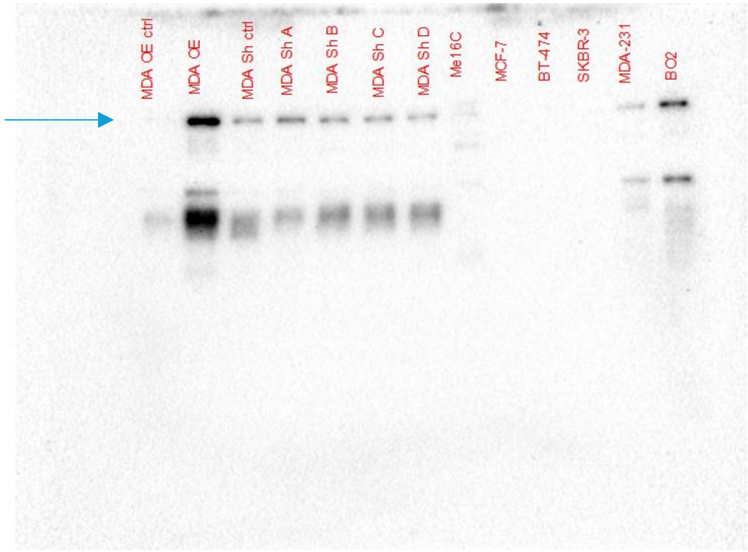

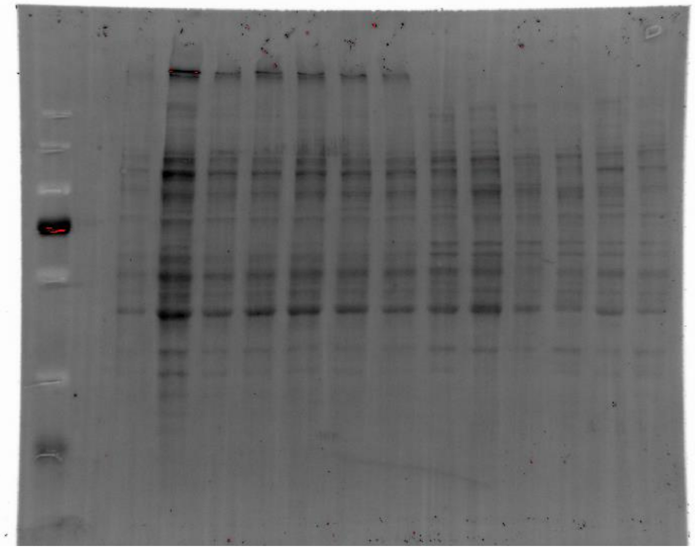

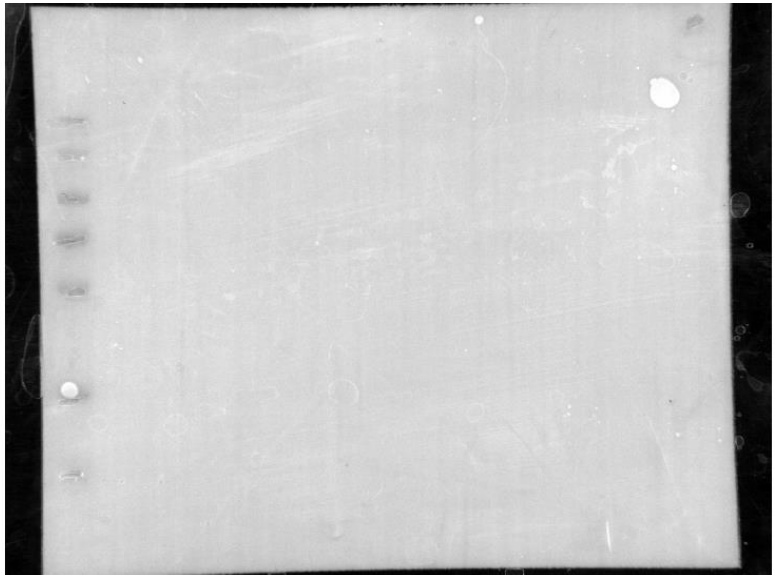

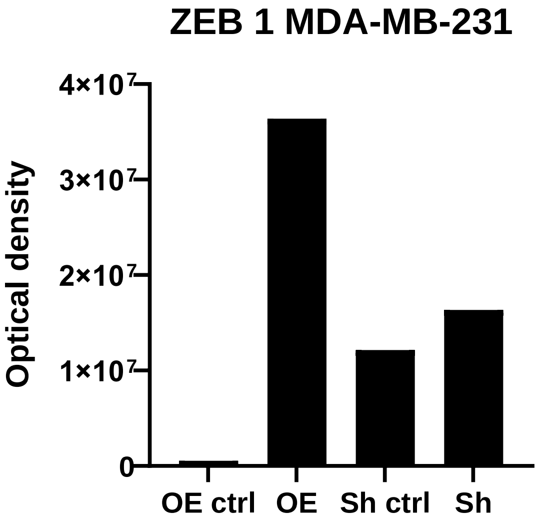

b) SKBR-3

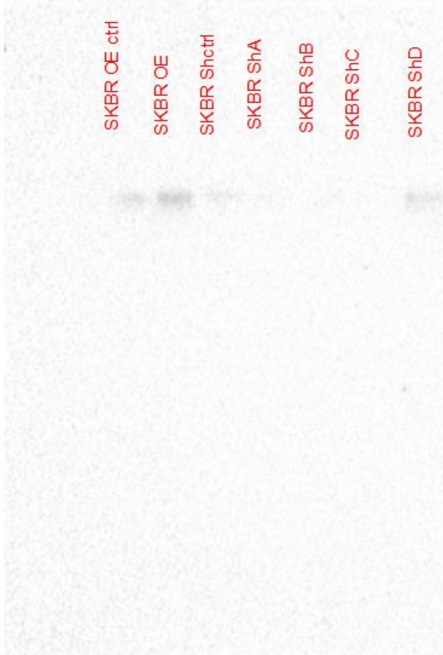
SKBR OE ctrl
SKBR OE
SKBR Shctrl
SKBR ShA
SKBR ShB
SKBR ShC
SKBR ShD

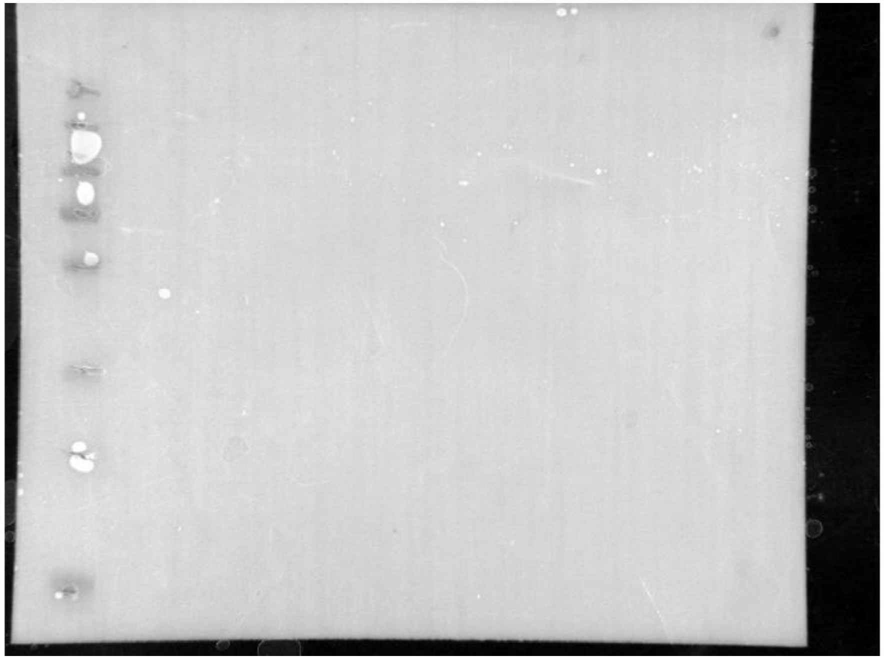

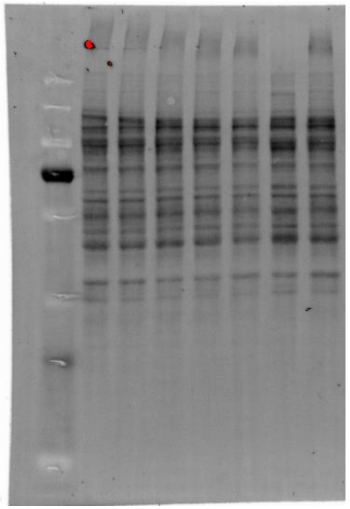

Supplement: Supplementary file 1 [file cells-13-01548-s001.zip › cells-3134991-supplementary.pdf]
